# Whole-brain modular dynamics at rest predict sensorimotor learning performance

**DOI:** 10.1162/netn_a_00420

**Published:** 2025-05-08

**Authors:** Dominic I. Standage, Daniel J. Gale, Joseph Y. Nashed, J. Randall Flanagan, Jason P. Gallivan

**Affiliations:** Department of Biomedical and Molecular Sciences, Queen’s University, Kingston, Canada; Centre for Neuroscience Studies, Queen’s University, Kingston, Canada; Department of Psychology, Queen’s University, Kingston, Canada

**Keywords:** Resting-state fMRI, Predictive markers, Dynamic modularity, Sensorimotor learning, Cognition

## Abstract

Neural measures that predict cognitive performance are informative about the mechanisms underlying cognitive phenomena, with diagnostic potential for neuropathologies with cognitive symptoms. Among such markers, the modularity (subnetwork composition) of whole-brain functional networks is especially promising due to its longstanding theoretical foundations and recent success in predicting clinical outcomes. We used functional magnetic resonance imaging to identify whole-brain modules at rest, calculating metrics of their spatiotemporal dynamics before and after a sensorimotor learning task on which fast learning is widely believed to be supported by a cognitive strategy. We found that participants’ learning performance was predicted by the degree of coordination of modular reconfiguration and the strength of recruitment and integration of networks derived during the task itself. Our findings identify these whole-brain metrics as promising network-based markers of cognition, with relevance to basic neuroscience and the potential for clinical application.

## Author Summary

The prediction of cognitive performance by measures derived from neuroimaging signals is informative about the neural processes underlying those signals, with the potential for use in the diagnosis of cognitive abnormalities, such as those associated with aging and Alzheimer’s disease. These *neural markers* include statistics of functional networks, characterizing the cooperation of distributed brain regions in large-scale networks. We investigated dynamics (changes over time) of functional networks prior to a task on which participants differ in their use of strategies. We found that performance on the upcoming task was predicted by several measures of whole-brain network dynamics.

## INTRODUCTION

Resting-state functional magnetic resonance imaging (rs-fMRI) is invaluable for investigating the brain’s intrinsic functional organization, free from explicit task engagement (Fox & Raichle, 2007; van den Heuvel & Pol, 2010). Resting-state functional connectivity (RSFC), determined by the pairwise covariation of hemodynamic signals at rest, has identified robust commonalities of this organization across large sets of participants (Buckner, Krienen, & Yeo, 2013) while simultaneously providing measures for exploring individual and group differences (Mueller et al., 2013; Vaidya & Gordon, 2013). These differences are the focus of intense research interest due to their predictive relationships with a wide variety of cognitive measures (Andrews-Hanna et al., 2007; Cole, Ito, Bassett, & Schultz, 2016; Ferreira & Busatto, 2013; van den Heuvel, Stam, Kahn, & Pol, 2009), neuropathologies (Contreras et al., 2019; Hull et al., 2017; Ibrahim et al., 2021; Mulders, van Eijndhoven, Schene, Beckmann, & Tendolkar, 2015; Pfannmöller & Lotze, 2019; Wilcox, Abbott, & Calhoun, 2019), and clinical interventions (Gallen & D’Esposito, 2019; Mehta et al., 2021; J. J. Taylor et al., 2021). As such, there is growing interest in RSFC measures that can account for behavioral variability across participant populations, with a view to identifying predictive biomarkers (Ferreira & Busatto, 2013; Gabrieli, Ghosh, & Whitfield-Gabrieli, 2015; Gallen & D’Esposito, 2019; Pfannmöller & Lotze, 2019; J. J. Taylor et al., 2021; Vaidya & Gordon, 2013; Wilcox et al., 2019). See Douw et al. (2019) for a thorough description of biomarkers.

A promising trend in this body of work is the predictive value of resting-state whole-brain modularity, or the subnetwork composition of functional brain networks at rest (Arnemann et al., 2015; Baniqued et al., 2018; Contreras et al., 2019; Gallen et al., 2016). Modularity conveys a number of advantages to complex systems, the gist of which is a balance between the competing demands of functional specialization and integration. The brain is highly modular, both structurally and functionally, conveying benefits to distributed information processing and emergent dynamics (Sporns & Betzel, 2016; see the Discussion section). As such, its candidacy as a predictor of cognitive performance is well founded.

Sensorimotor adaptation (SA) tasks are ideal for investigating individual differences in cognition, since participants show a high degree of variability in their use of cognitive strategies toward these tasks (de Brouwer, Albaghdadi, Flanagan, & Gallivan, 2018; Fernandez-Ruiz, Wong, Armstrong, & Flanagan, 2011; Standage et al., 2023). On SA tasks, an established sensorimotor mapping is systematically altered, such as visual feedback of hand motion while reaching, so that participants are required to learn a new mapping for competent task performance. A large body of data supports the hypothesis that learning on SA tasks is driven by two components, one implicit and one explicit (McDougle, Ivry, & Taylor, 2016). The implicit component is slow, nonvolitional, and widely believed to involve the recalibration of an internal model encoded by the motor system (Shadmehr, Smith, & Krakauer, 2010; Wolpert, Diedrichsen, & Flanagan, 2011). The explicit component is fast, volitional, and corresponds to the use of a strategy toward the task, such as aiming to the left of a bullseye to counteract a strong rightward wind (Haith, Huberdeau, & Krakauer, 2015; Morehead, Qasim, Crossley, & Ivry, 2015; J. A. Taylor, Krakauer, & Ivry, 2014).

We recently studied the whole-brain dynamics of SA, finding that cohesive or coordinated modular reconfiguration was associated with fast learning during the task and, by association, with the use of a cognitive strategy. We also derived task-based functional networks from the dynamic modules, finding subnetworks that were associated with fast learning during early adaptation and readaptation, respectively (Standage et al., 2023). Here, we refer to these networks as “the learning network” and “the relearning network” because of these behavioral associations. Importantly, our in-scanner SA task was preceded (and followed) by a resting scan, enabling us to investigate the extent to which spontaneous patterns of brain activity at rest are predictive of subsequent task performance.

We tested three principal hypotheses on resting-state, network-based markers of fast learning and, by association, cognitive performance. We hypothesized that fast learning among healthy young adults would be predicted by the degree of (1) dynamic modularity, (2) coordinated modular reconfiguration, and (3) recruitment and integration of task-derived networks. Hypothesis 1 was based on the aforementioned findings that static modularity at rest can predict training-based improvements in cognitive performance among patient groups (Arnemann et al., 2015) and healthy older adults (Baniqued et al., 2018; Gallen et al., 2016). Hypothesis 2 was based on our earlier finding that coordinated modular reconfiguration is “conducive” to a cognitive strategy toward adaptation, but does not directly implement that strategy (Standage et al., 2023). We reasoned that this conduciveness might also be observable at rest, prior to the task. Hypothesis 3 was based on our earlier finding that the learning and relearning networks are associated with fast adaptation. We reasoned that if recruitment of these (or other) task-based networks is supportive of task performance (and/or individual differences in performance; Standage et al., 2023), then a predisposition toward their composition would be advantageous. Conversely, we reasoned that if their integration is disruptive to task performance (Standage et al., 2023), then such a predisposition would be disadvantageous. All predictive measures were requantified after the task in a second resting scan, enabling us to determine whether participants’ intrinsic whole-brain networks were modified by adaptation, as seen with some other tasks and network measures (Albert, Robertson, & Miall, 2009; Lewis, Baldassarre, Committeri, Romani, & Corbetta, 2009; McGregor & Gribble, 2015).

## RESULTS

We analyzed rs-fMRI data from a study (Standage et al., 2023) in which participants performed a classic visuomotor rotation (VMR) task (Krakauer, 2009), controlling a small force sensor with the hand in order to move a cursor to a target that appeared randomly at one of eight locations around an invisible circle. On each of two testing days (separated by 24 hr), participants performed a baseline block, during which motion of the cursor was veridically mapped to motion of the hand, and a learning block, during which the correspondence between hand movement and cursor movement was rotated clockwise by 45°. This rotation required participants to adapt their movements (in the counterclockwise direction) to intercept the target. Resting scans were taken immediately before and after the task, during which participants lay still with their eyes open, maintaining central fixation. See the Methods section for details of the full experimental regimen.

Participants’ group-level behavior was typical of this class of task, as (on average) they reduced their errors during the learning block and also showed “savings” or faster relearning upon reexposure to the VMR on the second day (Figure 2A). However, these group-level data obscured significant interparticipant variability, as some participants were fast learners on each day (Figure 2B top), others were slow learners on each day (Figure 2B middle), and others were slow learners on the first day (Figure 2B bottom, magenta trace) and fast learners on the second day (cyan trace). To determine if these individual differences revealed a continuum of learning outcomes or a limited number of learning profiles, we clustered (see the Methods section) participants’ early error on Day 1 and Day 2, late error on Day 1 and Day 2, and savings (see caption to Figure 2). These five measures are commonly used to characterize behavior on VMR tasks (de Brouwer et al., 2018; Haith et al., 2015; Morehead et al., 2015; Standage et al., 2023), as they capture participants’ rate of learning (early error on each day), completeness of learning (late error on each day), and improvement in learning rate over the 2 days (savings), respectively.

**Figure 1.**
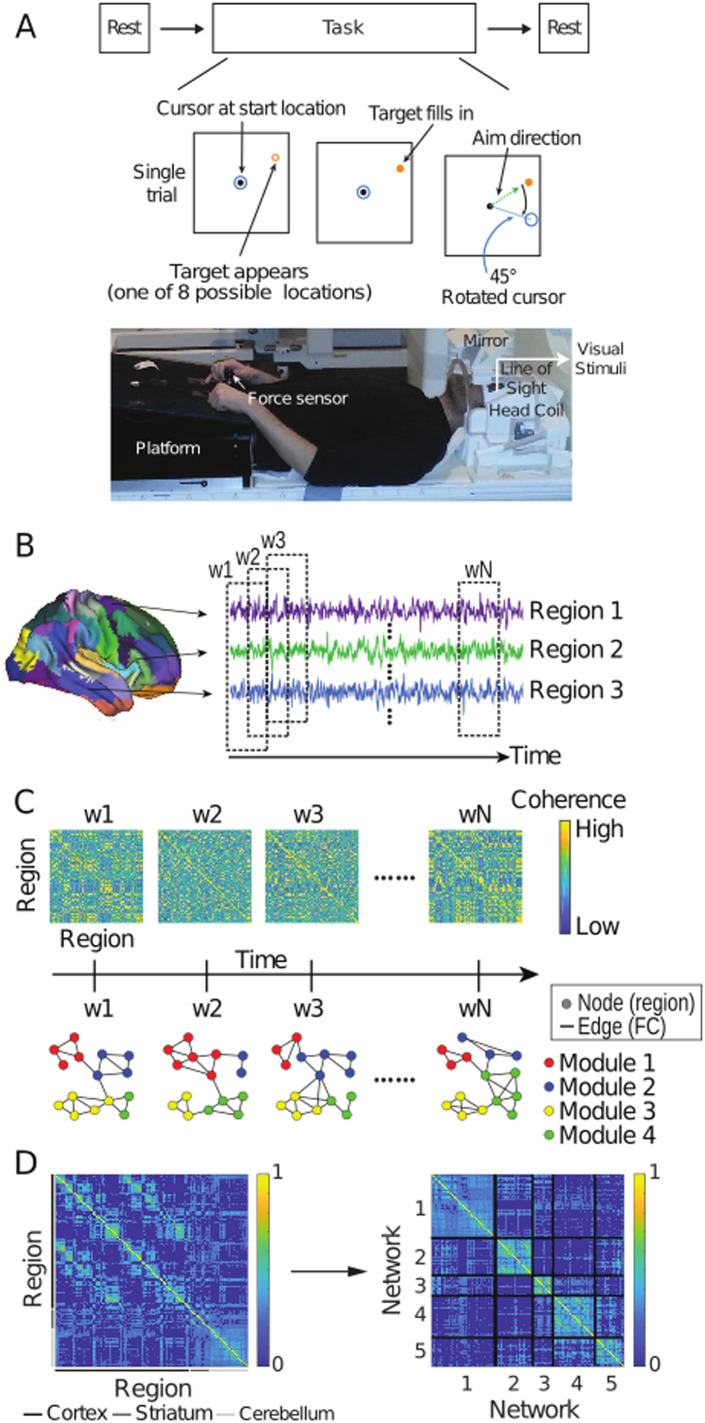
Overview of the task and neural analyses. (A) Participants underwent a resting scan before and after a VMR task, during which the viewed cursor, controlled by the hand, was rotated about the movement start location. (B) For each participant, the cerebral cortex, striatum, and cerebellum were parcellated into discrete regions and the average %BOLD time-series was extracted from each region during resting and task scans (three example cortical regions are shown). (C) The coherence of (Haar family) wavelet coefficients was calculated for each pair of regions in sliding time windows to construct functional connectivity matrices for each window (w1 – wN, see the Methods section). Time-resolved clustering methods were applied to the resulting multislice networks, identifying dynamic modules across time slices (four modules in the schematic). (D) Module allegiance matrix (left) showing the probability that each pair of brain regions was in the same module during early learning, calculated over all participants, modular partitions, and time windows (left). The arrow depicts the clustering of this matrix, identifying networks (right) that summarize the modular dynamics (see the Methods section). Brain plots of these networks are shown in Supporting Information Figure S1.

**Figure 2.**
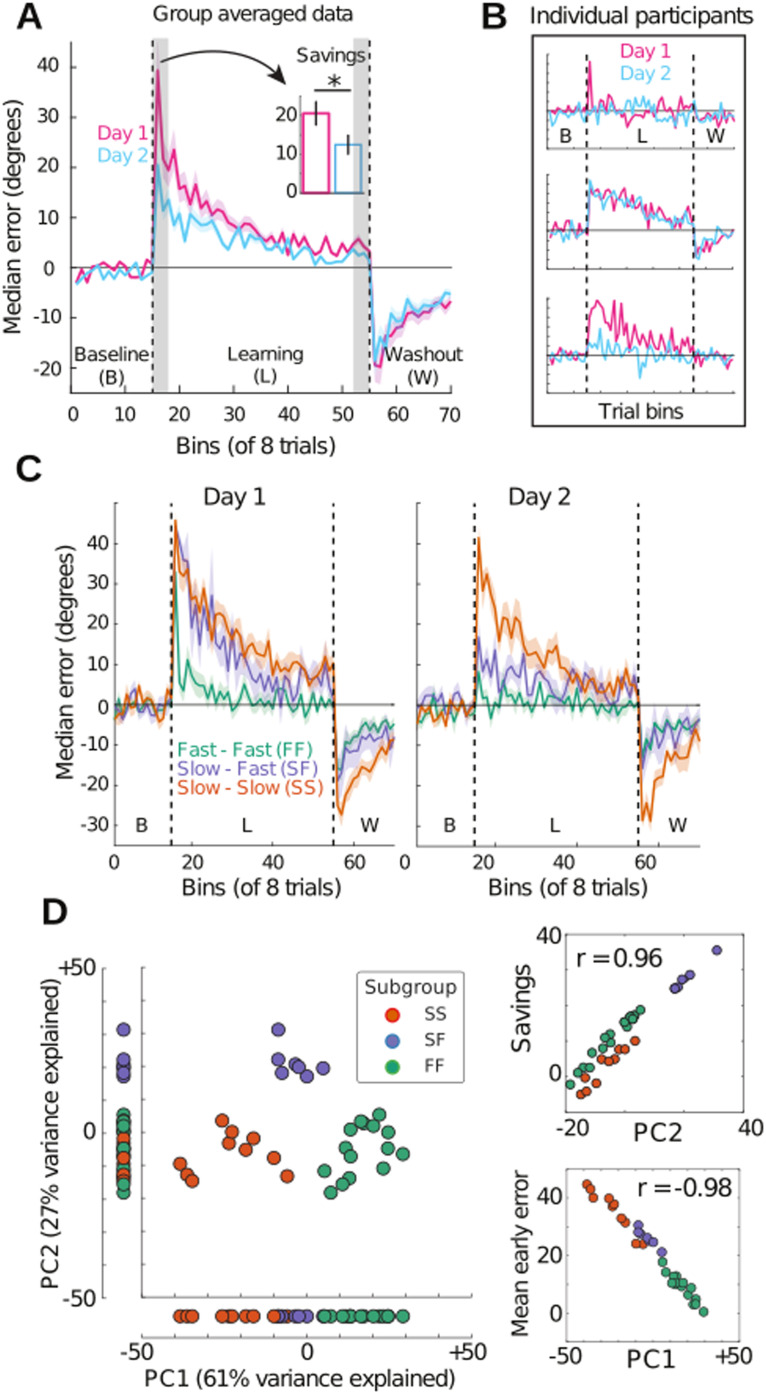
Participants were assigned to one of three subgroups by a clustering of their behavioral data. (A) Mean (across participants) of bin median errors during baseline (nonrotation), learning (45° rotation of the cursor), and “washout” (nonrotation, to unlearn the mapping prior to the second day) on Day 1 (pink) and Day 2 (cyan). Bins consisted of eight consecutive trials, during which the target was chosen at random without replacement from eight equidistant locations around an invisible circle. Early error and late error were defined as the first and last three learning bins, respectively (gray shading). Ribbons show standard error (±1 *SE*), and the dashed vertical lines demarcate the three task blocks. Savings (inset, early error on Day 1 minus early error on Day 2) was significant at the group level (paired *t* test; *t*[31] = 6.122, P = 8.666e-7). (B) The group-averaged approach in panel A obscures individual differences in learning. The trajectory of errors by three example participants shows a participant who learned quickly on both days (top, error is reduced quickly on Day 1 [magenta] and on Day 2 [cyan]), a participant who learned slowly on both days (middle, error is reduced slowly on both days) and a participant who learned slowly on Day 1 and quickly on Day 2 (bottom). (C) A clustering of participants’ early error on each day, late error on each day, and savings (five variables in total) identified three subgroups of participants whose mean bin median errors resemble the example participants in panel B on Day 1 (left) and Day 2 (right). We refer to these subgroups as FF (fast learning on both days), SS (slow learning on both days), and SF (slow learning on Day 1 and fast learning on Day 2). (D) Top two PCA components for the five learning measures across participants. Data points correspond to participants, color-coded by their cluster-assigned subgroup (legend in panel C). The horizontal axis shows that PC1 accurately classifies 31 of 32 participants (97% accuracy). Scatter plots on the right show that PC1 and PC2 closely correspond to mean early error across days and savings, respectively.

Our clustering revealed three distinct subgroups or “profiles” of learners (Figure 2C), exemplified by the individual participants shown in Figure 2B. These subgroups are well described as fast-fast (FF; fast learning on Day 1 and Day 2), slow-slow (SS; slow learning on Day 1 and Day 2), and slow-fast (SF; slow learning on Day 1 and fast learning on Day 2). Notably, the FF and SS subgroups clearly correspond to more explicit and implicit learners, respectively, whereas the SF subgroup is ambiguous in this regard (Standage et al., 2023). Due to issues of statistical power relating to the subgroup sizes (*N*[FF] = 15, *N*[SS] = 10, *N*[SF] = 7), we sought to identify a single scalar measure corresponding to subgroup membership, enabling us to investigate brain-behavior relationships with standard linear regression methods (prior to post hoc testing for subgroup differences). We therefore ran principal component analysis (PCA) on the five behavioral measures used to identify the subgroups. The first component (PC1) accounted for 61% of the variance in the data and accurately classified 31 out of 32 participants (Figure 2C). For intuitiveness, we reversed the sign of PC1, so that higher scores correspond to faster learning (and smaller errors). Of note, we found that the second principal component (PC2) closely corresponds to savings (*r* = 0.96; Figure 2D, upper inset) and accounts for 27% of the variance in the data. Because of its correspondence with savings, PC2 does not distinguish the FF and SS subgroups from one another (Figure 2D, vertical axis), so it is a poor “proxy” for subgroup membership. Therefore, PC2 does not feature in our subsequent analyses, but we wish to emphasize that the scatterplot of the first two principal components (Figure 2D) demonstrates that the subgroups correspond to highly distinct clusters in a space that accounts for 88% of the variance in the data, where the axes of this space correspond to interpretable measures of behavior. The interested reader can find the full behavioral analysis in Standage et al. (2023).

To test our hypotheses on dynamic modularity at rest and its efficacy as a predictor of upcoming task performance, we constructed “multislice” functional networks from the covariation of regional brain signals (in sliding time windows) during a resting scan immediately before (and after) the task. We partitioned these resting-state networks into spatiotemporal modules that maximized a quality function *Q* (Mucha, Richardson, Macon, Porter, & Onnela, 2010) and verified that participants’ whole-brain networks showed significant modularity (Bassett et al., 2011). Thus, we established that participants’ intrinsic functional networks were composed of interacting subnetworks before and after task performance (Supporting Information Figure S2).

### Contrary to Our Hypothesis, Modularity Scores at Rest Did Not Predict Learning Performance

**Figure 3.**
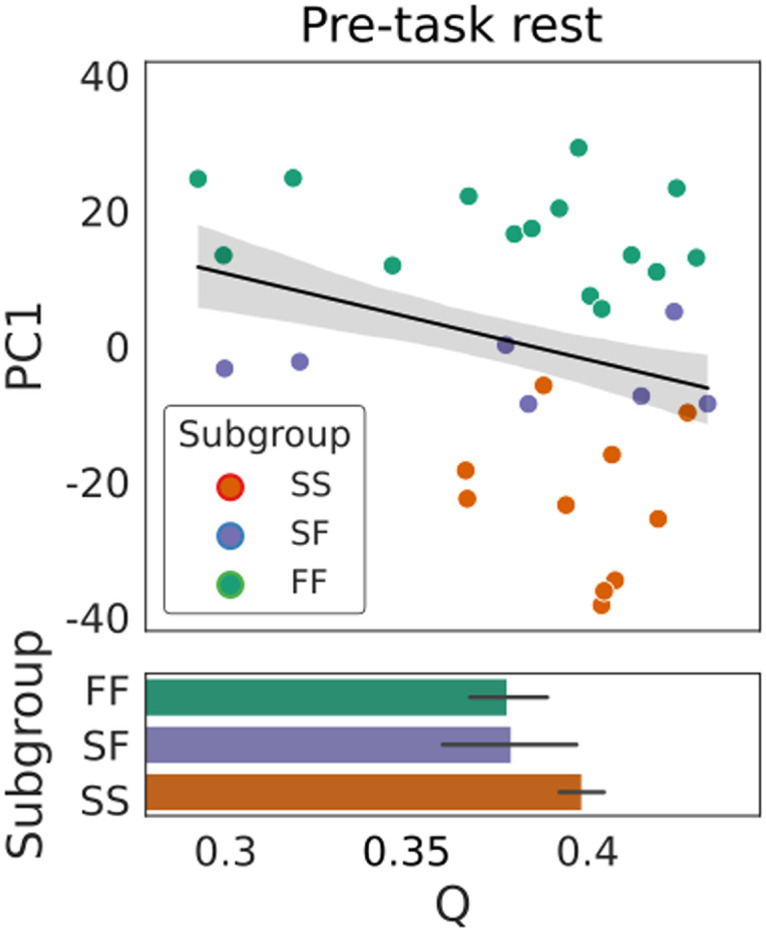
Prior to learning, greater dynamic modularity scores did not predict better task performance. The scatter plot shows PC1 for each subject, our proxy for behavioral subgroup membership, as a function of the mean quality function score Q (quantifying modularity, see text). Filled circles correspond to the FF, SS, and SF behavioral subgroups (see legend). The fitted line shows a linear regression model (least squares fit) where the shaded area corresponds to ±1 *SE*. Subgroup means ±1 *SE* are shown as bar graphs below the scatter plots. Contrary to our hypothesis, the fit was nonsignificant (*R*^2^ = 0.070, *F*[1, 30] = 2.246, *p* = 0.144).

To test our first hypothesis that higher dynamic modularity scores at rest would predict a faster learning profile over the 2-day task, we averaged the quality function score *Q* (Mucha et al., 2010) over all modular partitions for each participant (see the Methods section). We then estimated the degree to which this mean *Q* value predicted PC1 by fitting a linear regression model to these data (ordinary least squares) where *Q* was the predictor and PC1 was the dependent variable. Our hypothesis was rejected, as the predictive relationship was nonsignificant (Figure 3A). Thus, as participants’ whole-brain functional networks evolved over the pre-task resting scan, the clarity with which these networks could be decomposed into subnetworks did not predict participants’ learning profiles on a task on which fast learning is strongly associated with cognition (McDougle et al., 2016). This finding contrasts with those of earlier analyses of static (unchanging, nondynamic) functional networks, the modularity of which was predictive of the success of training regimens for cognitive improvements in patient groups and healthy older adults (Gallen & D’Esposito, 2019). We return to this contrast in the Discussion section.

### Coordinated Modular Reconfiguration at Rest Predicted Learning Performance

To test our second hypothesis that the coordination of modular reconfiguration at rest would predict participants’ learning profiles, we calculated the cohesive and disjointed flexibility (a.k.a. cohesion strength and disjointedness) of participants’ brain regions during the first resting scan. Cohesion strength refers to the number of times a given region changes modules together with each other region, summed over all other regions and divided by the number of transitions between time windows (the number of windows minus one). In contrast, disjointedness refers to the number of times a given region changes modules on its own, relative to the number transitions between time windows (Telesford et al., 2017). For each participant, we therefore calculated each of these measures for each brain region in each partition before averaging all partitions and regional scores (see the Methods section).

**Figure 4.**
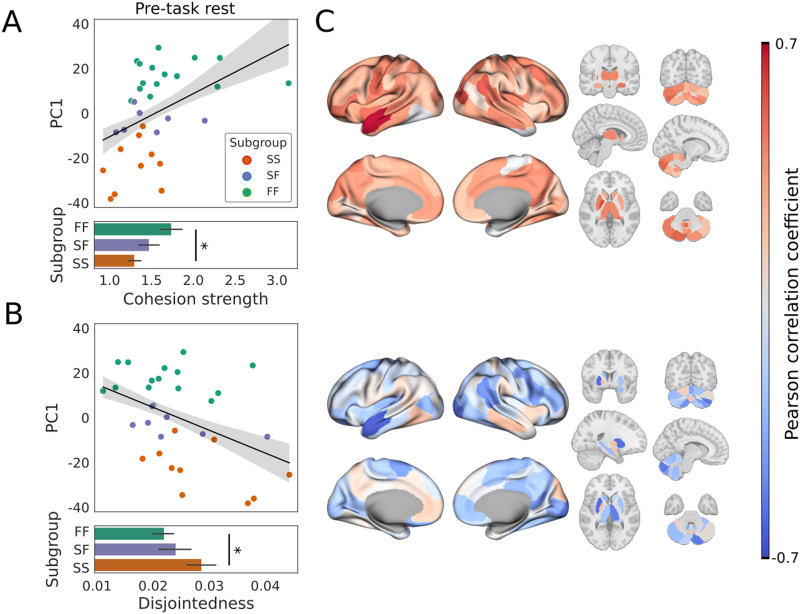
Coordinated (A) and uncoordinated (B) modular reconfiguration at rest predicted fast and slow learning profiles on the upcoming task, respectively. (A) PC1 as a function of mean cohesive flexibility (cohesion strength, see text). The fit of a linear regression model (black line; gray shading shows ±1 *SE*) showed a positive predictive relationship between cohesion strength (the predictor) and PC1 (*R*^2^ = 0.198, *F*[1, 30] = 7.401, *p* = 0.011), where FF was more cohesive than SS (two-sample *t* test: *t*[23] = 2.518, *p* = 0.019), but SF did not differ significantly from FF (*t*[20] = 1.233, *p* = 0.232) or SS (*t*[15] = 1.176, *p* = 0.258). (B) PC1 as a function of mean disjointed flexibility (disjointedness, see text). The fit of a linear regression model (black line) showed a negative predictive relationship between disjointedness (the predictor) and PC1 (*R*^2^ = 0.186, *F*[1, 30] = 6.852, *p* = 0.014), where SS was more disjointed than FF (*t*[23] = −2.089, *p* = 0.048), but SF did not differ significantly from SS (*t*[15] = −1.115, *p* = 0.282) or FF (*t*[20] = −0.596, *p* = 0.558). (C) Brain plots show Pearson’s correlation coefficient ($|r|=\sqrt{R^{2}}$) for cohesion strength and PC1 (upper plots) and disjointedness and PC1 (lower plots) for individual brain regions. Correlation coefficients associated with cohesion strength (mostly positive) and disjointedness (mostly negative) are shown with a divergent color scheme, ranging from strongly negative (dark blue) to strongly positive (dark red). In panels A and B, subgroup means ±1 *SE* are shown as bar graphs below the scatter plots, where stars indicate significant differences (*p* < 0.05).

Consistent with our hypothesis, the fit of a linear regression model showed cohesion strength to be a significant predictor of PC1, where cohesion strength was stronger among the FF subgroup than the SS subgroup, but did not differ statistically between either of these subgroups and SF (Figure 4A). We determined the model’s internal validity with leave-one-out cross validation (LOOCV), calculating the squared difference between the real and predicted node strength for each participant when the model was trained on the other participants’ predictor and dependent variables (mean squared error = 339.572, standard deviation = 353.342). We then determined the statistical significance of this performance metric by permuting the mapping between these variables 10,000 times, producing a null distribution of (across-participant) mean squared errors (see the Methods section “Linear Regression Modeling”). The model was significant under this approach (*p* = 0.020).

Inversely, the predictive relationship between disjointedness (the predictor) and PC1 was significantly negative, where disjointedness was stronger among the SS subgroup than FF, but did not differ statistically between either of these subgroups and SF (Figure 4B). This prediction was internally validated by permutation testing with LOOCV, using the same procedure as for cohesion strength above (mean squared error = 336.556, standard deviation = 397.982, *p* = 0.017). As was the case during early learning (Standage et al., 2023), both measures of modular reconfiguration captured global, whole-brain dynamics, as the predictive relationship between cohesion strength and PC1 was positive for 138 out of 142 brain regions, whereas the relationship between disjointedness and PC1 was negative for 112 out of 142 regions (Figure 4C). It is worth noting that cohesion strength and disjointedness are not opposites, but they are not formally independent either. In a given condition, they can be expected to have a negative relationship because a greater number of regions moving together from one module to another leaves fewer regions (and directed pairs of modules) available for independent movement. Nonetheless, their respective relationships with codependent variables are not redundant. Earlier studies have shown significant differences (in relation to task variables) for cohesion strength and not disjointedness (Telesford et al., 2017) and for disjointedness and not cohesion strength (Malagurski, Liem, Oschwald, Mérillat, & Jäncke, 2020; Telesford et al., 2017). Furthermore, it is possible for these (mean) measures to be positively correlated, as was the case for 13 individual brain regions in our study. The correlation between mean cohesion strength and disjointedness was strongly negative here (*r* = −0.718; Supporting Information Figure S3), but we include both measures to provide an intuitive and thorough account of modular reconfiguration.

### Resting-State Recruitment and Integration of Task-Based Networks Predicted Learning Performance

To test our hypothesis that the recruitment of task-relevant networks at rest would predict participants’ learning profiles, we assigned each brain region to one of five task-based networks derived during early learning (Supporting Information Figure S1; Standage et al., 2023), when the use of cognitive strategies toward the task has been shown to be most pronounced (supporting the fastest learning; McDougle, Bond, & Taylor, 2015; J. A. Taylor et al., 2014). The “recruitment” of an individual network can be measured in terms of the “interaction” between two networks by calculating the network’s interaction with itself (Bassett, Yang, Wymbs, & Grafton, 2015). Under this approach, the interaction between two networks is defined by *I*_*k*1, *k*2_ = (∑*i* ∈ *C*_*k*1_, *j* ∈ *C*_*k*2_*P*_*i, j*_)/(∣ *C*_*k*1_∣∣*C*_*k*2_∣ ), where *C*_*k*∈{1,2}_ are modules, ∣*C*_*k*_∣ is the number of regions they contain, and *P*_*i, j*_ is a “module allegiance matrix” quantifying the probability that each pair of regions was in the same module (here, during the first resting scan). Under this approach, recruitment is therefore measured by letting *k*1 = *k*2, quantifying the consistency of regional interactions within a specified network over time.

**Figure 5.**
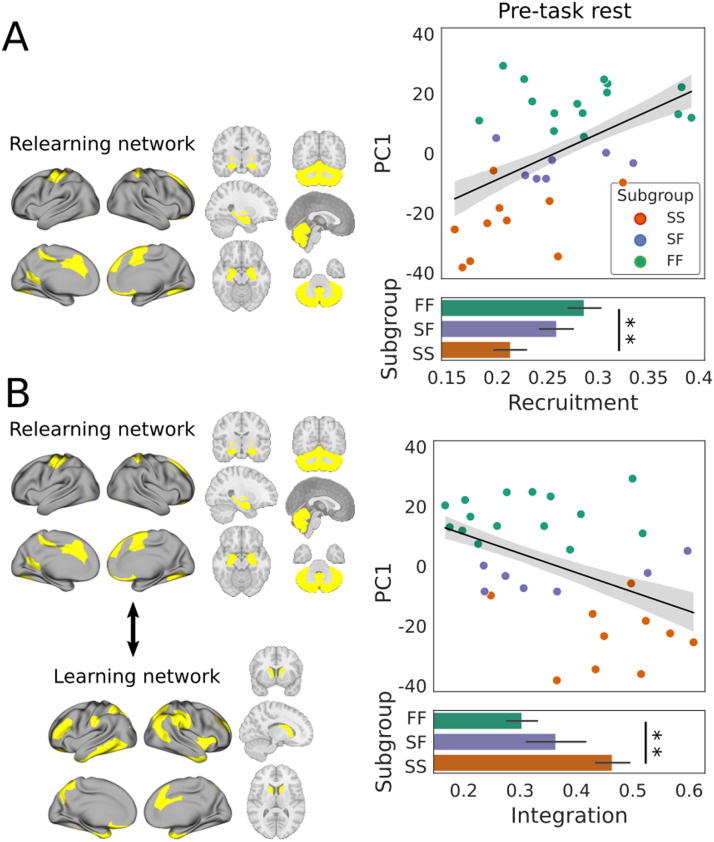
Prior to learning, stronger recruitment of the relearning network, and weaker integration between the learning and relearning networks, predicted faster learning and thereby a more strategic (cognitive) approach to the task. (A) The relearning network (left) consisted of regions spanning contralateral motor cortex, bilateral cerebellum, medial prefrontal cortex, and several subcortical structures (bilateral hippocampus, pallidum, amygdala, and accumbens). Recruitment of this network at rest was a significant predictor of PC1, as determined by the fit of a linear regression model (black line; *R*^2^ = 0.253, *F*[1, 30] = 10.15, *p* = 0.003, FDR-adjusted *p* = 0.017). Gray shading shows ±1 *SE*. Recruitment by FF was stronger than SS (*t*[23] = 3.028, *p* = 0.006), but recruitment by SF did not differ statistically from that of FF (*t*[20] = 1.013, *p* = 0.323) or SS (*t*[15] = 1.858, *p* = 0.083). (B) The learning network (left) consisted of regions spanning the anterior temporal pole, inferior and superior parietal, dorsolateral prefrontal cortex, and the bilateral caudate. Its pre-task integration with the relearning network was (negatively) predictive of PC1 (*R*^2^ = 0.199, *F*[1, 30] = 7.447, *p* = 0.010), where integration was stronger by the SS subgroup than FF (*t*[23] = 3.574, *p* = 0.002) but did not differ statistically between SF and FF (*t*[20] = 1.061, *p* = 0.301) or SS (*t*[15] = −1.673, *p* = 0.115). Error bars show ±1 *SE*. In bar plots, two stars indicate significant differences (*p* < 0.01).

One at a time, we fit a linear regression model to the recruitment of each of the five networks (serving as the predictor variable) and PC1 (the dependent variable). Following false discovery rate (FDR) correction over the five networks (see the Methods section), the relearning network was the only network whose recruitment predicted PC1 (Figure 5), where the prediction was validated by permutation testing with LOOCV (mean squared error = 339.569, standard deviation = 352.094, *p* = 0.020). This finding was surprising to us since the relearning network was not associated with participants’ behavioral profiles during the task until the *second* day of the experiment (Standage et al., 2023). As was the case with modular reconfiguration above, the FF and SS subgroups were significantly different according to this measure (FF recruited the network more strongly than SS), but neither of these subgroups differed statistically from SF (Figure 5A).

Next, we sought to determine whether integration of the relearning network with any of the other four task-based networks would predict participants’ behavioral profiles. The integration between two networks *k*1 ≠ *k*2 can be measured by the normalized interaction between them $I_{k1,k2}^{\prime }=I_{k1,k2}/\sqrt{\left(I_{k1,k1}I_{k2,k2}\right)}$ (Bassett et al., 2015). As such, integration quantifies the degree to which networks interact over time, relative to their size. Following FDR correction over the other four networks, we found a negative predictive relationship between integration of the learning and relearning networks (the predictor) and PC1 (*R*^2^ = 0.199, *F*[1, 30] = 7.447, *p* = 0.011, corrected *p* = 0.042) where the prediction was validated by permutation testing with LOOCV (mean squared error = 325.482, standard deviation = 407.276, *p* = 0.009). The two networks were significantly more integrated among the SS subgroup than FF, but neither of these subgroups differed statistically from SF (Figure 5B).

Because we measured recruitment and integration at rest, prior to learning, but the five networks underlying these measurements were derived during learning itself, we wondered if our results would hold if summary networks were derived at rest. In this regard, we reasoned that if pre-task recruitment conveyed a performance advantage on the actual task (as evidenced by its prediction of PC1), then the recruited network should be sufficiently distinguishable to be derived before the task. We therefore applied our network-derivation procedure (Standage et al., 2023) to the first resting scan. Notably, the procedure identified four networks, not five (see Supporting Information Figure S4), implying that modular reconfiguration during the task entailed the construction of new, task-appropriate functional networks. We then identified the network derived during rest that was most similar to the relearning network (derived during early learning), where similarity was determined by the Jaccard index (Jaccard, 1912), that is, the similarity of two networks was quantified as the size of their intersection divided by the size of their union (multiplied by 100). We then calculated the recruitment score for the most similar resting-state summary network (Jaccard index = 76%). Crucially, the results of this analysis replicated the recruitment results above. That is, during the first resting scan, recruitment of our “resting-state relearning network” predicted PC1 (*R*^2^ = 0.250, *F*[1, 30] = 9.975, *p* = 0.004, corrected *p* = 0.014), where this prediction was validated by permutation testing with LOOCV (mean squared error = 309.228, standard deviation = 381.823, *p* = 0.005). Recruitment was stronger by the FF subgroup than SS (*t*[23] = 2.918, *p* = 0.008), but did not differ significantly between SF and FF (*t*[20] = 0.503, *p* = 0.621) or SS (*t*[15] = 1.724, *p* = 0.105). These results not only indicate that the regions of the relearning network were sufficiently coupled during pre-task rest to be distinguishable as a network at that time, but they also confirm that recruitment of this resting-state network was predictive of subsequent learning performance. For completeness, we calculated the recruitment score for this network during early relearning on Day 2 of the task, when the relearning network distinguished the FF and SF subgroups from the SS subgroup (Standage et al., 2023). Once again, we replicated our results, as we found a significant positive correlation between recruitment and PC1 (*r* = 0.450, *p* = 0.010, corrected *p* = 0.039), where recruitment was stronger by the FF (*t*[23] = 2.412, *p* = 0.024) and SF (*t*[15] = 3.179, *p* = 0.006) subgroups than the SS subgroup, but did not differ significantly between FF and SF (*t*[20] = −0.157, *p* = 0.877). This stringent test shows that this network was not only predictive of performance on the upcoming task, but also that it could “stand in” for the relearning network during relearning itself.

Following these results, we sought to determine if PC1 was predicted by integration of the above “resting-state relearning network” with a “resting-state learning network.” The latter, however, appears to be highly task specific, as all four resting-state networks were at least 59% similar to one of the other four task-based networks, but no single resting-state network was more than 20% similar to the learning network derived during adaptation. At first glance, this finding may appear quizzical, in light of our (above) result that integration of the learning and relearning networks during rest predicts slow learning (Figure 5B). There is nothing quizzical about it, however. We assigned task-based network labels (from 1 to 5) to brain regions and then calculated the recruitment and integration of each group of labeled regions at rest. It turns out that the learning network had not been “put together” prior to the task. This finding indicates that slower learning was actually predicted by greater integration of its *dispersed* regions with the relearning network. In other words, the learning network was not recruited until the task was performed, but we were still able to calculate the integration of its regions (distributed across multiple networks) with the relearning network. This measure of integration predicted slow learning (Figure 5B).

### The Strength of RSFC Predicted Learning Performance

**Figure 6.**
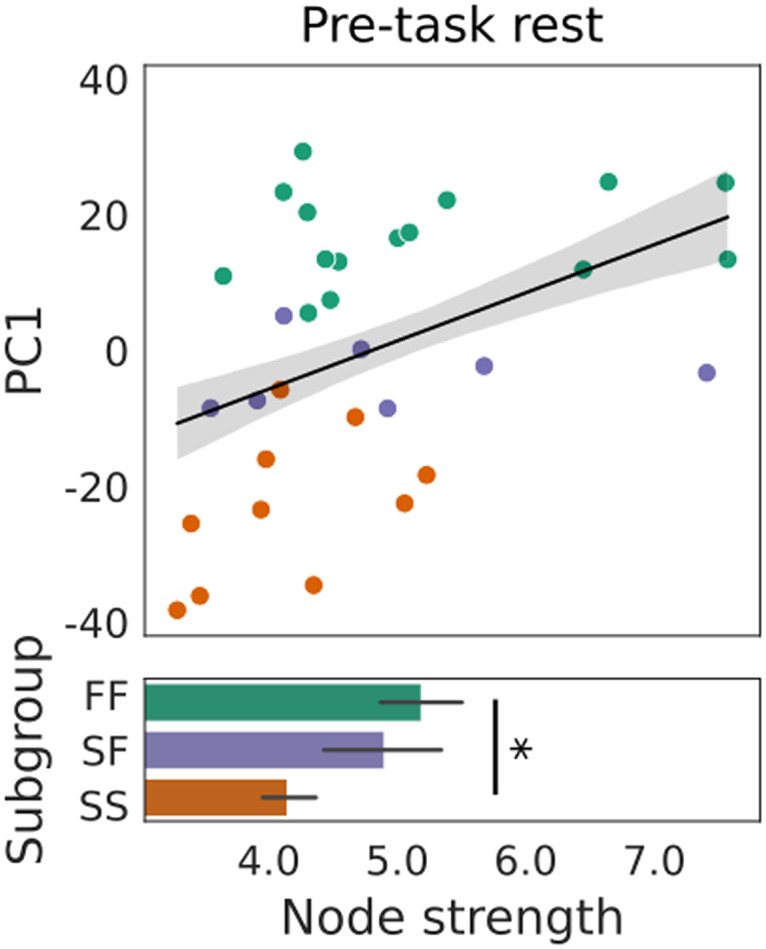
Prior to learning, stronger RSFC predicted better task performance. The scatter plot shows PC1 as a function of node strength (mean strength of connectivity over all brain regions over all temporal windows for each subject), where the black line shows the significant fit of linear regression model +1 *SE* (*R*^2^ = 0.188, *F*[1, 30] = 6.968, *p* = 0.013). Node strength was stronger among the FF subgroup than the SS subgroup (two-sample *t* test: *t*[23] = 2.368, *p* = 0.027), but did not differ statistically between FF and SF (*t*[20] = 0.488, *p* = 0.631) or SS and SF (*t*[15] = 1.556, *p* = 0.141). The star in the bar plot indicates statistical significance (*p* < 0.05).

The above findings demonstrate that modular dynamics during pre-task rest predict performance on a task where fast learning is believed to be supported by a cognitive strategy (McDougle et al., 2016). To better understand these findings, we turned our attention to the functional connectivity from which the dynamic modules were derived in the first place, asking whether stronger RSFC (i.e., stronger coherence between the activation of brain regions) could provide the foundation for the (putatively) beneficial dynamics exhibited by faster learners. We therefore estimated the degree to which the mean node strength (for each node, the sum of connection strengths) predicted PC1 by fitting a linear regression model to these data. Node strength did indeed predict PC1 (*R*^2^ = 0.188, *F*[1, 30] = 6.793, *p* = 0.013), where the prediction was validated by permutation testing with LOOCV (mean squared error = 332.139, standard deviation = 335.699, *p* = 0.014). Faster learners had stronger pre-task connectivity (Figure 6), where node strength was greater among the FF subgroup than the SS subgroup, but did not differ statistically between either of these subgroups and SF (Figure 6). To confirm that this finding was not an artifact of significance thresholding in network construction (weaker networks were sparser), we reran our analysis with uniformly sparse networks, replicating our results (and results above; Supporting Information Figure S5).

In light of this finding, we sought to better understand the joint effect of node strength and modular dynamics on behavior. To this end, we calculated the correlation between each pair of successful predictor variables above (cohesion strength, disjointedness, recruitment, integration, and node strength), finding that several of them are strongly correlated (Supporting Information Figure S3). We therefore ran PCA on the five predictors, fitting a multiple regression model to the first five principal component scores and PC1 (the dependent variable). The overall fit was significant (*R*^2^ = 0.359, adjusted *R*^2^ = 0.236, *F*[5, 26] = 2.914, *p* = 0.032), and the coefficient corresponding to the first component was significantly different from zero (component 1: *t*[31] = 3.600, *p* = 0.001; component 2: *t*[31] = 0.971, *p* = 0.340; component 3: *t*[31] = 0.224, *p* = 0.825; component 4: *t*[31] = 0.243, *p* = 0.810; component 5: *t*[31] = 0.745, *p* = 0.463). This component loaded most strongly onto node strength, explaining 62% of the variance in the data, though it loaded similarly onto other predictor variables (cohesion strength: −0.491; disjointedness: 0.464, recruitment: −0.409, integration: 0.331, node strength: −0.516). We return to this finding in the Discussion section.

### Resting-State Recruitment and Integration of Task-Based Networks Differed Before and After Learning

Having tested our hypotheses on predictive, network-based markers of cognition, we sought to determine whether resting-state modular dynamics would differ before and after task performance. That is, we asked whether whole-brain dynamics at rest were altered by learning. To answer this question, we calculated whole-group differences (Rest 2 minus Rest 1) for the six measures tested above for their predictive relationships with PC1, that is, for *Q*, cohesion strength, disjointedness, recruitment of the relearning network, integration of the relearning network with (the dispersed regions of) the learning network, and node strength. Following FDR correction over the six tests, none of these differences were significant (paired *t* test, *Q: t*[31] = 0.718, *p* = 0.478, corrected *p* = 0.717; cohesion strength: *t*[31] = −0.117, *p* = 0.908, corrected *p* = 0.973; disjointedness: *t*[31] = 0.953, *p* = 0.348, corrected *p* = 0.696; recruitment: *t*[31] = 2.420, *p* = 0.022, corrected *p* = 0.066; integration: *t*[31] = 2.732, *p* = 0.010, corrected *p* = 0.060; and node strength: *t*[31] = −0.034, *p* = 0.973, corrected *p* = 0.973).

**Figure 7.**
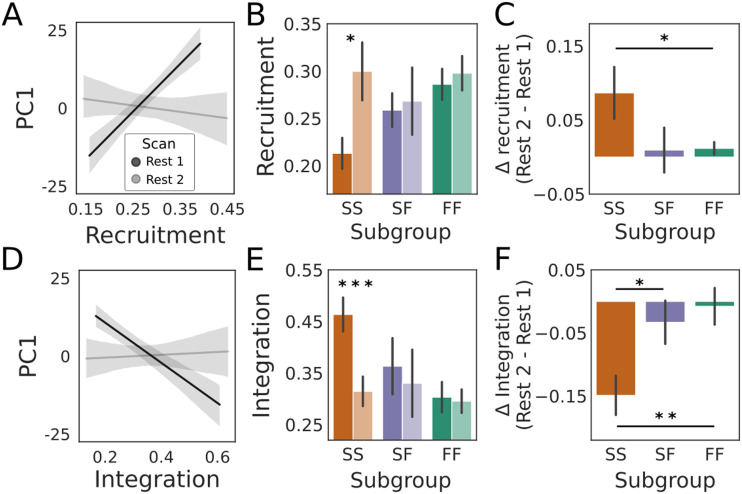
(A) The Pearson correlation between recruitment of the relearning network and PC1 was stronger (Steiger’s test for dependent correlations: *t*[30 ] = 3.636 *p* = 0.001, corrected *p* = 0.005) during the first (pre-task) resting scan (Rest 1, black: *r* = 0.501, *p* = 0.003; *r* = $\sqrt{R^{2}}$ per Figure 5A) than the second (post-task) resting scan (Rest 2, gray: *r* = −0.087, *p* = 0.635). (B) The change in recruitment was driven by the SS subgroup, whose recruitment was significantly greater (paired *t* test: *t*[9] = 2.475, *p* = 0.035) during the second resting scan (lighter shade) than the first (darker shade). Neither of the FF (*t*[14] = 1.386, *p* = 0.187) or SF (*t*[6] = 0.311, *p* = 0.766) subgroups differed significantly between resting scans. (C) The change in recruitment by SS was significantly greater than that by FF (two-sample *t* test: *t*[23] = −2.487, *p* = 0.021) but did not differ significantly between SS and SF (*t*[15] = 0.096, *p* = 0.924) or between SF and FF (*t*[20] = −1.573, *p* = 0.137). (D) The correlation between integration (of the relearning network with the regions of the learning network) and PC1 was weaker (Steiger’s test for dependent correlations: *t*[30 ] = −3.136, *p* = 0.004, corrected *p* = 0.010) during the first resting scan (Rest 1, black, *r* = −0.446, *p* = 0.010; ∣r∣ = $\sqrt{R^{2}}$ per Figure 5B) than the second resting scan (Rest 2, gray, *r* = 0.025, *p* = 0.890). (E) The change was again driven by the SS subgroup, whose integration was significantly weaker (*t*[9] = −4.798, *p* = 9.758e-4) during the second resting (lighter shade) scan than during the first (darker shade). The FF (*t*[14] = −0.266, *p* = 0.794) and SF (*t*[6] = −0.964, *p* = 0.372) subgroups did not differ significantly between resting scans. (F) The change in integration by SS was significantly greater than that by SF (two-sample *t* test: *t*[15] = −2.468, *p* = 0.027) and FF (*t*[23] = 3.230, *p* = 0.004), but FF and SF were not significantly different (*t*[20] = 0.520, *p* = 0.609). In all panels, stars indicate statistical significance (one star: *p* < 0.05; two stars: *p* < 0.01; three stars: *p* < 1e-3).

To determine whether the relationship between each of these measures and PC1 was different during the second resting scan (compared with the first resting scan), we calculated the Pearson correlation coefficient between each measure and PC1 during each scan. For each measure, we then used Steiger’s test (a.k.a. Williams’ test and Meng’s test) for dependent correlations (Steiger, 1980) to determine if the correlation differed. Following FDR correction over the five tests, the correlation between recruitment of the relearning network and PC1 was significantly different after learning (Figure 7A). We therefore calculated the difference in recruitment for each subgroup, finding a significant increase during post-task rest among SS participants, where this increase was significantly greater than the change in recruitment by FF participants (Figure 7B). The correlation between integration (of the learning and relearning networks) and PC1 was also significantly different following FDR correction (Figure 7C), where we found a significant decrease during post-task rest among SS participants, the magnitude of which was significantly greater than the change in integration by FF and SF participants (Figure 7D). Thus, these changes in intrinsic network composition were driven by the SS subgroup, which recruited the relearning network more strongly in the second resting scan, relative to the first, and disentangled it from the dispersed regions that formed the learning network during the task. However, this reorganization does not appear to have influenced the behavior of SS participants on the second day. In other words, resting-state recruitment and integration of the learning and relearning networks by SS became more like those of FF and SF after learning, but these changes did not predict improved performance the next day. We return to these findings in the Discussion section.

### The Organization of Dynamic Functional Connectivity Was More Similar Between Resting Scans Than Between Either Resting Scan and Learning

**Figure 8.**
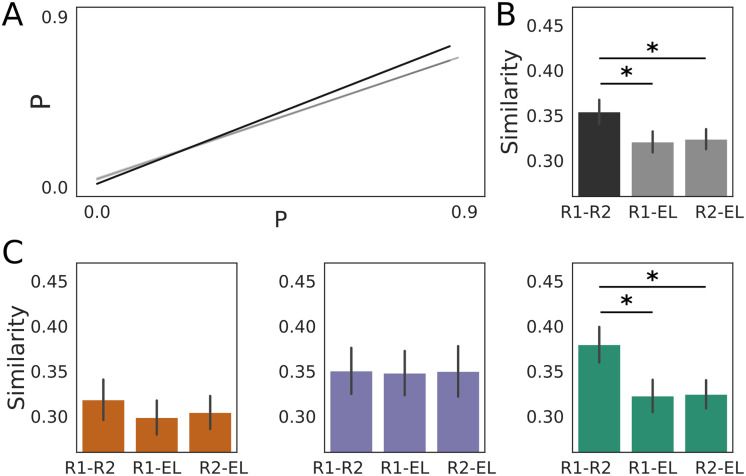
The organization of dynamic, resting-state functional networks was more similar during pre-task (Rest 1) and post-task (Rest 2) rest than when either resting scan was compared with learning. (A) Linear fit to whole-group module allegiance probabilities (P, the probability that each of the 10,011 pairs of brain regions were in the same module; see text) for Rest 1 and Rest 2 (black, *r* = 0.838), Rest 1 and early learning (gray, *r* = 0.765), and Rest 2 and early learning (gray, *r* = 0.761). The gray lines are nearly indiscernible, as is the standard error in all fits. Fitted data points are omitted for clarity. (B) Mean similarity (Pearson’s *r*) of module allegiance matrices across all participants for each pair of scans (R1: Rest 1; R2: Rest 2: EL: early learning). Rest 1 and Rest 2 were significantly more similar than Rest 1 and early learning (paired one-tailed *t* test: *t*[31] = 2.174, *p* = 0.019) and Rest 2 and early learning (R2-EL; *t*[31] = 1.918, *p* = 0.032), whereas the correlation between Rest 1 and early learning was not statistically different from Rest 2 and early learning (paired two-tailed *t* test: *t*[31] = −0.269, *p* = 0.790). Note that our use of one-tailed *t* tests reflects our testing of predictions in panel “A.” (C) Mean similarity of each pair of scans for each behavioral subgroup (left, SS; middle, SF; right, FF). Among FF participants, module allegiance matrices from the resting scans were more similar than Rest 1 and early learning (paired one-tailed *t* test: *t*[14 ) = −2.112, *p* = 0.027) or Rest 2 and early learning (*t*[14] = −2.574, *p* = 0.011), whereas the resting scans’ correlations with learning were not significantly different (paired two-tailed *t* test: *t*[14] = 0.108, *p* = 0.542). Among SF and SS participants, Rest 1 and Rest 2 were not significantly more similar than Rest 1 and early learning (paired one-tailed *t* test, SF: *t*[6] = −0.069, *p* = 0.474; SS: *t*[9] = −1.648, *p* = 0.067) or Rest 2 and early learning (SF: *t*[6] = −0.015, *p* = 0.494; SS: *t*[9] = −0.489, *p* = 0.318) and the correlation between Rest 1 and early learning was not statistically different from Rest 2 and early learning (paired two-tailed *t* test, SF: *t*[6] = 0.102, *p* = 0.539; SS: *t*[9] = 0.241, *p* = 0.592). Single stars in panels B and C indicate statistical significance (*p* < 0.05).

Our derivation of four summary networks from pre-task rest (compared with the five networks derived from early learning) suggests a fundamental difference between the organization of dynamic, whole-brain functional networks at rest and during the task. In light of this finding, we predicted that the module allegiance matrices from which the networks were derived would be more similar when the resting scans were compared with each other than when either resting scan was compared with early learning. To test this prediction, we calculated the pairwise correlations between module allegiance matrices from these three scans. All three correlations were strong (Rest 1 [pre-task] and Rest 2 [post-task]: Pearson’s *r* = 0.838, *p* ≈ 0; Rest 1 and early learning: *r* = 0.765, *p* ≈ 0; Rest 2 and early learning: 0.761, *p* ≈ 0), where Rest 1 and Rest 2 were more similar (more highly correlated) than Rest 1 and early learning (Steiger’s test for dependent correlations: *t*[30] = 20.508, *p* ≈ 0) and Rest 2 and early learning (*t*[30] = 21.777, *p* ≈ 0). Notably, the correlation between Rest 1 and early learning did not differ significantly from that between Rest 2 and early learning (*t*[30] = 1.262, *p* = 0.207). Thus, our prediction was confirmed for module allegiance matrices taken over all participants (Figure 8A). To bolster these findings, we calculated the same correlations for each pair of scans for each participant, testing the means (across participants) for the above relationships. This approach corroborated our findings, as the resting scans were again significantly more similar to each other than either of them was to early learning (Figure 8B). Among the behavioral subgroups, these findings were the case for FF, but not for SF and SS. Thus, the whole-group effect was driven by FF (Figure 8C).

## DISCUSSION

Neural predictors of cognition and learning are the subject of growing research interest (Ferreira & Busatto, 2013; Gabrieli et al., 2015; Gallen & D’Esposito, 2019; Pfannmöller & Lotze, 2019; J. J. Taylor et al., 2021; Vaidya & Gordon, 2013; Wilcox et al., 2019). Within this body of work, the modularity of static functional networks at rest has been shown to be a promising measure for predicting neuropathologies and their responsiveness to treatment programs (Gallen & D’Esposito, 2019). Based on these findings, and on recent studies of the modular dynamics of cognition and learning (Standage et al., 2023), we asked whether dynamic modularity at rest would predict group differences on a task with well-characterized behavioral variability, on which fast learning is widely believed to reveal the use of a cognitive strategy (McDougle et al., 2016). First, we tested the hypothesis that modularity itself (the degree to which brain regions cluster together in functional subnetworks over time) would predict the learning profiles of participants who were fast learners on both days (FF), slow learners on both days (SS), and slow learners on the first day and fast learners on the second day (SF). At rest, prior to task performance, the modularity score *Q* did not predict a fast learning profile on the upcoming task (Figure 3). Second, we tested the hypothesis that the degree of coordination of modular reconfiguration would predict participants’ learning profiles, finding that FF and SS participants could be distinguished by the cohesiveness and disjointedness of modular changes at rest (Figure 4). Third, we tested the hypothesis that the recruitment and integration of functional networks derived during early learning, when the use of cognitive strategies is commonly believed to be most pronounced (McDougle et al., 2015; J. A. Taylor et al., 2014), would predict participants’ learning profiles. We not only found that recruitment of the relearning network at rest predicted fast adaptation, but we also found that its integration with (the regions of) the learning network predicted slow adaptation, even though the learning network itself was not constructed until participants performed the task. Both measures distinguished the FF and SS subgroups from one another (Figure 5). To ground these findings in the functional networks from which the temporal modules were derived, we tested the hypothesis that the strength of RSFC would predict participants’ learning profiles, finding that FF and SS participants could be distinguished by node strength (Figure 6). Overall, these findings are consistent with the hypothesis that modular dynamics offer a promising set of network-based markers of the propensity for cognition, due to individual differences in the functional connectivity from which they are derived.

Of course, the propensity for cognition before an experimental task does not imply cognitive ability in a general sense, and we do not claim that measurements of dynamic modularity can (or should) be used to assess intelligence. For example, participants with more coordinated modular reconfiguration may have been more alert and task-ready than their peers, owing to any number of factors, such as the relative demands of their schedules, sleep, stress, or caffeine consumption. Nonetheless, our predictive measures (except *Q*) did distinguish fast learners from slow learners on the upcoming task, where fast learning is widely believed to be supported by the use of a cognitive strategy (McDougle et al., 2016). Thus, we are comfortable claiming to have provided evidence that these measures reveal the conduciveness of participants’ whole-brain dynamics to the implementation of strategies. As such, our results speak to participants’ cognitive states, but they may not speak to cognitive traits (see below).

A related issue is the directionality of our resting-state predictions. We were able to distinguish preidentified fast learners from slow learners by measures of modular dynamics at rest, but we did not assign participants to the subgroups based on these measures. Furthermore, all measures used here predicted fast learning relative to slow learning or vice versa, but it would be premature to propose a threshold for the use of these statistics, above or below which individuals may be classified as belonging to one group or another. Thus, despite the promise of our findings, it is unclear if measures of modular dynamics are candidates for use in clinical diagnosis. Modular analysis of pooled resting-state data would help to determine the “normal range” of modular measures.

### Most of Our Hypotheses Were Confirmed, But Some Results Were Surprising

We hypothesized that dynamic modularity scores during pre-task rest would predict participants’ learning profiles on an SA task. This hypothesis was largely based on predictive relationships between static modularity and cognition in training regimens for older adults (Gallen & D’Esposito, 2019), as well as our own work associating modular dynamics with performance during the SA task (Standage et al., 2023). There are many differences between these earlier studies and the present one that may explain the empirical rejection of our hypothesis, perhaps most notably the age and cognitive health of participants in our study (healthy, 18–35 years old; see the Methods section). Additionally, the very focus of dynamic modularity is network reconfiguration, whereas static modularity abstracts over these dynamics. These two approaches to quantifying subnetwork composition are not expected to align across all conditions, though we are unaware of systematic comparisons in this regard. By definition, they are more likely to align in more stable dynamic regimes, and clearly, the utility of static measures is questionable in unstable regimes. For a review of dynamic approaches to rs-fMRI, see Lurie et al. (2020). Future work should consider these and other explanations of this negative result.

Our hypotheses on cohesion strength and disjointedness were borne out in the data, as each of these resting-state measures predicted learning outcomes in the expected manner. Specifically, greater cohesion strength (Figure 4A) predicted fast learning and greater disjointedness predicted slow learning (Figure 4B). Our hypothesis that participants’ learning profiles would be predicted by resting-state recruitment and integration of task-based networks was also borne out, but we do not claim that our results on recruitment and integration were as expected. Other things being equal, we might have expected pre-task recruitment of the learning network to predict task performance, since this network was linked to fast learning on the same day (Standage et al., 2023). However, as described above, we were unable to derive the learning network during pre-task rest, indicating that its constituent regions began working together as a network during the task itself. Nevertheless, the more that its dispersed regions were integrated with the relearning network, the more likely participants were to be slow learners (Figure 5B). While this finding may not be considered surprising per se, we did not anticipate it. We were more surprised that recruitment of the relearning network predicted fast learning (Figure 5A) despite its inability to distinguish the subgroups during task performance until the next day (Standage et al., 2023). Since the networks were derived during the task on Day 1, it seems likely that all participants were using this network in some way, but their use of it did not differentiate the subgroups until Day 2, when (putatively) it was involved in recalling earlier strategies.

We were also surprised that the SS subgroup recruited the relearning network more strongly during post-task rest than pre-task rest (Figure 7). As such, SS participants’ organization of their intrinsic networks became more like that of FF and SF participants, but this similarity with their faster learning peers did not result in faster learning on the second day. Other things being equal, we might have expected the SF subgroup to show this sort of learning-induced change, since SF performance improved so dramatically on Day 2. In this regard, it is worth noting that we only investigated learning-induced changes in the measures for which we had pre-task hypotheses in the first place (see the Introduction section). It is possible (perhaps likely) that other intrinsic network measures change after task performance, such that these changes would be informative about SF participants’ Day 2 improvement. Future work should address this possibility.

With respect to recruitment and integration more generally, it is worth reiterating that we derived the summary networks during the task (Standage et al., 2023) before quantifying their recruitment and integration during pre- and post-task rest. This approach contrasts with the more common one of measuring some aspect of preidentified resting-state networks during task performance (e.g., Gale et al., 2022; Vatansever, Menon, Manktelow, Sahakian, & Stamatakis, 2015). Our approach is both philosophical and practical. The typical use of resting-state networks for task-based analyses leverages a common currency of sorts, assuming that the composition and properties of functional networks among a small number of people (on a variety of tasks) can be understood in terms of the composition and properties of intrinsic networks derived from a large number of people (Gordon et al., 2016; Hutchison & Gallivan, 2018; Schaefer et al., 2018; Thomas Yeo et al., 2011). This approach looks forward from a general predisposition to a specific outcome. The use of task-based networks for resting-state analyses is more tightly focused, looking backward from a specific outcome to a specific predisposition (among the same participants whose RSFC is under study). We believe these two approaches should be seen as complementary. Practically, our focus was the predictive value of modular dynamics during pre-task rest, so from this perspective, it made sense to use networks derived from dynamic modules linked to performance during the upcoming task.

Our finding that mean node strength predicted participants’ learning profiles is interesting in its own right, but it warrants comment in relation to other predictive variables in our study. Firstly, studies linking the strength of RSFC to cognition typically select sets of connections from whole-brain networks, based on their strong correlations with behavioral variables of interest (Finn et al., 2015; Rosenberg et al., 2016). As such, our use of a global (average) measure of RSFC strength to predict participants’ behavioral profiles is appealing for its generality, bearing in mind that our predictions are internally validated only. Secondly, this generality may have implications for modular dynamics. Here, it is worth emphasizing the stages of our analysis pipeline, in which node strength can be considered a first-order measure, derived directly from dynamic RSFC; cohesion strength and disjointedness can be considered second-order measures, derived from modular partitions that were in turn derived from RSFC; and recruitment and integration can be considered third-order measures, derived from allegiance matrices (Figure 1) that were derived from modular partitions. While we cannot claim that coordinated modular reconfiguration requires strong global connectivity, we wish to emphasize that dynamic RSFC provides the underlying foundation for the higher order predictors in our study and that we observed a strong correlation between node strength and cohesion strength (Pearson’s *r* = 0.903; Supporting Information Figure S3). These points of emphasis suggest that stronger functional connectivity provides a better foundation for the coordinated modular dynamics associated with task-based cognition (Standage et al., 2023). If so, it may be that task engagement renders the strength of participants’ global functional connectivity more uniform, so that the relationship between its strength and modular dynamics is more evident during the resting state. If so, then these predictors may provide an index into readiness for cognitive engagement. Future work should address this possibility. Our present intuition is that stronger functional networks provide a platform for more coordinated reconfiguration, in turn facilitating more efficient recruitment of task-relevant networks.

### Networks Derived at Rest Differed From Those Derived During Learning

Our derivation of four summary networks from each of the first and second resting scans (Supporting Information Figure S4) was conspicuously different from the five networks derived during early learning (Standage et al., 2023). This difference was especially pronounced in relation to the learning network, which was no more than 20% similar to any network derived from either resting scan (quantified by the Jaccard index). This finding is consistent with the hypothesis that whole-brain cognitive control involves the “putting together” of functional networks to suit the task at hand (Standage et al., 2023). Here, participants recruited (put together) the learning network during the SA task, pulling its regions from all of the four pre-task resting-state networks, except for the “resting-state relearning network.” Since all of these networks (rest and task) were derived from module allegiance matrices summarizing modular dynamics in a given scan (Bassett et al., 2015), we used these matrices to quantify the similarity of modular organization in each pair of scans. In this regard, we found that pre- and post-task resting states were more similar to one another than to early learning, which was (statistically) equally similar to both of these resting scans (Figure 8A–B). This whole-group finding was driven by the FF subgroup, as it was not the case for the SF and SS subgroups (Figure 8C). This result too is consistent with the hypothesis that cognitive control corresponds to recruitment of functional networks to suit the task at hand, since the FF subgroup was the only one whose behavior implied a strategic approach to the task on Day 1. More generally, these findings suggest that the organization of resting-state networks before and after tasks may be informative about the whole-brain mechanisms underlying task performance, including individual and group differences. Further work should investigate this intriguing possibility.

### Methodological Considerations

At least three methodological considerations warrant further comment. Our first consideration is the very use of dynamic modularity and its statistics as predictors of cognition. To begin with, modularity is a network measure. While early work in this area demonstrated the predictive properties of individual brain regions, these findings were not robust across studies (Gallen & D’Esposito, 2019), suggesting that a common frame of reference across large groups of participants should be the first criterion for a neural marker. If so, then the second criterion should be individual and group variation within that framework. RSFC provides this balance of competing factors (Buckner et al., 2013). It is broadly accepted that cognitive phenomena are supported by large-scale network interactions (Bressler & Menon, 2010; Smallwood et al., 2021; Zabelina & Andrews-Hanna, 2016), so it makes sense to consider large-scale networks for cognitive predictors. Modularity confers many benefits to large-scale networks by balancing functional specialization with integration over specialized subsystems (Wig, 2017). Most notably for the present work, modularity supports adaptability, enabling more efficient (Tosh & McNally, 2015) and robust (Ellefsen, Mouret, & Clune, 2015) learning. As such, modularity (quantified by *Q* here) appears to be an ideal network-based marker for predicting the success of learning regimens for overcoming cognitive deficits, such as those associated with brain injury (Arnemann et al., 2015) and old age (Gallen et al., 2016). Nonetheless, the relationship between *Q* (which did not predict behavior), modular reconfiguration, and subnetwork recruitment is not straightforward. It is plausible that modular dynamics constitute a whole-brain trait that requires coordination among regions to produce qualitative changes in whole-brain states, supporting the recruitment of functional networks. This characterization is consistent with the association of deeper (shallower) states of unconsciousness with more disjointed (cohesive) modular reconfiguration (Standage et al., 2020), but further research is needed to understand such large-scale network dynamics. Whole-brain models and formal analysis of their emergent dynamics are an exciting direction in this regard (Breakspear, 2017).

A second consideration is our use of scale 1 (Haar) wavelet coefficients to construct coherence networks from participants’ BOLD timeseries. Scale 1 corresponds to a frequency band of 0.12–0.25 Hz, which is higher than usual for fMRI analysis (e.g., 0.06–0.12 Hz; Bassett et al., 2015; Standage et al., 2023). We did not have an a priori reason to analyze the data in this band, but rather, we felt that our hypotheses were sufficiently well founded (see the Introduction section) to test them at scale 1 when they were clearly refuted by visual observation at scale 2. Had most of our scale 1 results not been so clearly aligned with our main hypotheses (all except *Q*), we would not have pursued them further, but we do not believe this alignment to be coincidental. In this regard, we wish to reiterate that our task-based networks were derived at scale 2 (Standage et al., 2023) and their resting-state recruitment and integration were calculated at scale 1 here (Figure 5). In the Results section “Coordinated Modular Reconfiguration at Rest Predicted Learning Performance”, we derived a resting-state approximation of the relearning network at scale 1 and replicated our resting-state recruitment findings. We also replicated our task results at scale 2 (Standage et al., 2023) with this “resting-state relearning network.” These stringent tests of robustness give us a high degree of confidence in our results, and they suggest that information content in fMRI timeseries may be present at a higher (or broader) range of frequencies for resting-state scans than is typically considered and, more intriguingly, that this content may be present at a higher frequency in the resting state than during tasks. In our view, it is potentially insightful that task-based hypotheses should be grounded in lower frequency analyses than their resting-state confirmation. The notion that brain dynamics at rest (e.g., dynamics supporting imaginary perceptions) may differ from those during tasks (e.g., sensory perceptions) is not new (Koenig-Robert & Pearson, 2021), but to the best of our knowledge, evidence that such different dynamics are manifest in metabolic differences revealed by magnetic resonance imaging is novel. Thus, our findings fit within a larger research effort to understand the mechanistic relationships between different frequencies of BOLD fluctuations (Keilholz et al., 2020; Yuen, Osachoff, & Chen, 2019), and we believe our approach and results provide a valuable addition to this emerging literature. For several decades, the content of BOLD timeseries at around 0.2 Hz has largely been ignored, due to respiratory artifacts at this frequency, but the presence of such artifacts does not preclude the presence of other content (e.g., DeRamus et al., 2021; Trapp, Vakamudi, & Posse, 2018; Yuen et al., 2019) and our preprocessing pipeline for artifact mitigation conforms to current standards of rigor (the Methods section “fMRI Preprocessing”).

A third consideration is our use of PC1 to identify predictive relationships between neural variables and participants’ learning profiles. While our use of PC1 in this way is well justified (Standage et al., 2023), this approach will only identify neural variables of interest where SF is sandwiched between FF and SS (or is roughly equal to either of them) since PC1 corresponds to mean early error across days (Figure 2D). For cohesion strength and disjointedness, this issue did not matter, as our hypotheses were confirmed. For recruitment and integration, however, we looked for relationships between PC1 and the recruitment of each of five networks, and between PC1 and the integration of the relearning network with the other networks (both sets of tests were corrected for multiple comparisons, described in the Results section). Had we looked for predictive relationships between neural variables and PC2 (which corresponds to savings; Figure 2D), we may have identified differences between SF and the other two subgroups, given SF’s pronounced improvement in learning performance on Day 2. Confirmation of each of our hypotheses clearly differentiated the FF and SS subgroups, which were composed of unambiguously fast (explicit) and slow (implicit) learners. The SF subgroup is (and remains) something of a curiosity. We leave this consideration to a follow-up study.

### Summary and Conclusions

Modularity is a promising network-based neural marker for cognitive pathologies and their responsiveness to clinical programs (Gallen & D’Esposito, 2019). Where most earlier work focused on static modularity (Arnemann et al., 2015; Baniqued et al., 2018; Gallen et al., 2016), quantifying the subnetwork composition of functional networks that average out the dynamics of a resting-state activity, we featured these dynamics by quantifying the subnetwork composition of functional networks over time (see also Gifford et al., 2020). By characterizing the time-evolution of this composition, we have demonstrated that standard measures of modular dynamics (Bassett et al., 2015; Standage et al., 2020, 2023; Telesford et al., 2017) at rest are able to predict learning profiles on a task with well-characterized individual differences (de Brouwer et al., 2018; Fernandez-Ruiz et al., 2011; Standage et al., 2023). These differences are widely believed to reveal the degree to which participants take a strategic (cognitive) approach to the task (McDougle et al., 2016), so our results highlight modular dynamics as a more general, network-based cognitive marker. They further highlight the promise of dynamic modularity in the clinical domain, with a view to diagnosis and treatment of cognitive pathologies.

## METHODS

### Experimental Design and Statistical Analysis

Forty right-handed individuals between the ages of 18 and 35 years (mean = 22.5, standard deviation = 4.51; 13 males) participated in the study and received financial compensation for their time. Data from eight participants were excluded, due to either head motion in the MRI scanner (*N* = 4; motion greater than 2 mm or 2° rotation within a single scan) or missing volumes in a large portion of one of the task scans (*N* = 4), leaving 32 participants in the final analysis. Right handedness was assessed with the Edinburgh Handedness Questionnaire (Oldfield (1971). Participants’ written, informed consent was obtained before commencement of the experimental protocol. The Queen’s University Research Ethics Board approved the study, which was conducted in accordance with the principles outlined in the Canadian Tri-Council Policy Statement on Ethical Conduct for Research Involving Humans and the principles of the Declaration of Helsinki (1964).

Experimenters were not blind to testing. Statistical significance was defined by *α* = 0.05. In all figures, error bars indicate ±1 standard error of the mean. All statistical tests were two tailed. FDR correction for family-wise error rate used the method by Benjamini and Hochberg (1995).

### Apparatus

In the scanner, participants performed hand movements that were directed toward a target by applying a directional force onto an MRI-compatible force sensor (ATI technologies) using their right index finger and thumb. The target and cursor stimuli were rear-projected with an LCD projector (NEC LT265 DLP projector, 1024 × 768 resolution, 60-Hz refresh rate) onto a screen mounted behind the participant. The stimuli on the screen were viewed through a mirror fixed to the MRI coil directly above participants’ eyes, thus preventing participants from being able to see the hand. The force sensor and associated cursor movement were sampled at 500 Hz.

### Procedure

The experiment used a well-established VMR task (Krakauer, 2009) to probe SA (Figure 1A). At the start of each trial, the cursor (20-pixel radius) appeared in a central position (25-pixel radius). A white target circle (30-pixel radius) was presented on a black screen, appearing at one of eight locations (0, 45, 90, 135, 180, 225, 270, 315°) on an invisible ring around the central position (300-pixel radius) and filled in (white) following a 200-ms delay. Once filled in, participants applied a brief directional force to the sensor (threshold of 1.5 N), which launched the cursor toward the target. Target location was drawn at random from the above locations without replacement, in bins of eight trials. Once launched, the cursor travelled the 300-pixel distance to the ring over a 750-ms period (with a bell-shaped velocity profile) before becoming stationary at the ring to provide participants with end-point error feedback. If the cursor overlapped the target to any extent, the target would become green, signifying a “hit.” Each trial was separated by 4 s, and within this period, participants had 2.6 s from the target presentation to complete the trial (including the 200-ms target delay, participants’ reaction time, and the 750-ms cursor movement; any remaining time was allotted to the end-point error feedback). At 2.6 s, the trial was ended, the screen blanked, and the data saved, and participants briefly waited for the next trial to begin. Reaction times were not emphasized. On trials in which the reaction time exceeded 2.6 s, the trial ended, and the participant waited for the next trial to begin. These discarded trials were rare (0.56% across all trials and participants) and were excluded from behavioral analyses. They were kept in the neuroimaging analysis due to the continuous nature of the fMRI task and our focus on functional connectivity.

During each testing session, 120 baseline trials (15 bins of 8 trials) were completed without a rotation of the cursor. Following these trials, 320 learning trials (40 bins of 8 trials) were completed, during which a 45° clockwise rotation of the cursor was applied. The baseline and learning trials were completed during one continuous fMRI scan. Following this scan, conditions were restored to baseline (i.e., no rotation of the cursor) in a separate scan and participants performed 120 washout (unlearning) trials. These washout trials allowed us to probe participants’ rate of relearning 24 hr later (and thereby their savings). In addition to these VMR-related task components, we interspersed three 6-min resting fMRI scans before, between, and after VMR learning and washout. During resting scans, participants were instructed to rest with their eyes open, while fixating a central cross presented on the screen. The total testing time was 75 min on each testing day.

### MRI Acquisition

Participants were scanned using a 3-Tesla Siemens TIM MAGNETOM Trio MRI scanner located at the Centre for Neuroscience Studies, Queen’s University (Kingston, Ontario, Canada). fMRI volumes were acquired using a 32-channel head coil and a T2*-weighted single-shot gradient-echo EPI acquisition sequence (TR = 2,000 ms, slice thickness = 4 mm, in-plane resolution = 3 mm × 3 mm, TE = 30 ms, field of view = 240 mm × 240 mm, matrix size = 80 × 80, flip angle = 90°, and acceleration factor [integrated parallel acquisition technologies] = 2 with generalized auto-calibrating partially parallel acquisitions reconstruction. Each volume comprised 34 contiguous (no gap) oblique slices acquired at a 30° caudal tilt with respect to the plane of the anterior and posterior commissure, providing whole-brain coverage of the cerebrum and cerebellum. A T1-weighted Alzheimer's Disease Neuroimaging Initiative magnetization prepared rapid gradient echo [ADNI MPRAGE] anatomical was also collected (TR = 1760 ms, TE = 2.98 ms, field of view = 192 mm × 240 mm × 256 mm, matrix size = 192 × 240 × 256, flip angle = 9°, 1 mm isotropic voxels). For each resting scan, 180 imaging volumes were collected. For the baseline and learning epochs, a single, continuous scan was collected of 896 imaging volumes duration. For the washout scan, one scan of 256 imaging volumes was collected. Each scan included an additional eight imaging volumes at both the beginning and end of the scan.

On Day 1, a separate practice session was carried out before the actual fMRI experiment to familiarize participants with the apparatus and task. This session involved performing 60 practice baseline trials. The fMRI testing session for each participant lasted 2 hr and included setup time (20 min), practice (10 min), one high-resolution anatomical scan (8 min), two diffusion tensor imaging scans (one in the anterior-to-posterior direction and the other in the posterior-to-anterior direction; 10 min), a resting scan (6 min), a baseline and rotation scan (30 min), a resting scan (6 min), a washout scan (9 min), and a final resting scan (6 min). In the present paper, we only consider fMRI data from the first and second resting scans, and the baseline-learning scan, on the first day.

### MRI Preprocessing

Preprocessing was performed with fMRIPrep 1.4.0 (Esteban et al., 2019, 2020; RRID:SCR_016216).

#### Anatomical data preprocessing.

T1w scans in each session were corrected for intensity nonuniformity (INU) with N4BiasFieldCorrection (Tustison et al., 2010), distributed with ANTs 2.2.0 (Avants, Epstein, Grossman, & Gee, 2008). The T1w-reference was then skull-stripped with a Nipype implementation of the antsBrainExtraction.sh workflow (from ANTs), using OASIS30ANTs as target template. Brain tissue segmentation of cerebrospinal fluid (CSF), white matter, and gray matter was performed on the brain-extracted T1w using fast (FSL 5.0.9; Zhang, Brady, & Smith, 2001). A T1w-reference map was computed after registration of the individual T1w scans (after INU-correction) using mri_robust_template (FreeSurfer 6.0.1; Reuter, Rosas, & Fischl, 2010). Brain surfaces were reconstructed using recon-all (FreeSurfer 6.0.1; Dale, Fischl, & Sereno, 1999), and the estimated brain mask was refined with a custom variation of the method to reconcile ANTs-derived and FreeSurfer-derived segmentations of the cortical gray matter of Mindboggle (Klein et al., 2017). Volume-based spatial normalization to FSL’s MNI ICBM 152 nonlinear 6th Generation Asymmetric Average Brain Stereotaxic Registration Model (Evans, Janke, Collins, & Baillet, 2012; TemplateFlow ID: MNI152NLin6Asym) was performed by a nonlinear registration with antsRegistration (ANTs 2.2.0), using brain-extracted versions of both T1w reference and the T1w template.

#### Functional data preprocessing.

For each BOLD run (for each participant), a reference volume and its skull-stripped version were generated using a custom methodology with fMRIPrep. The BOLD reference was then coregistered to the T1w reference using bbregister (FreeSurfer), implementing boundary-based registration (Greve & Fischl, 2009). Coregistration was configured with 9 degrees of freedom to account for distortions remaining in the BOLD reference. Head-motion parameters with respect to the BOLD reference (transformation matrices, and six corresponding rotation and translation parameters) were estimated before any spatiotemporal filtering using mcflirt (FSL 5.0.9; Jenkinson, Bannister, Brady, & Smith, 2002). BOLD runs were slice-time corrected using 3dTshift from AFNI 20160207 (Cox & Hyde, 1997; RRID:SCR_005927). The BOLD time-series were resampled into standard space (MNI152NLin6Asym), generating spatially normalized, preprocessed BOLD runs. A reference volume and its skull-stripped version were generated using a custom methodology with fMRIPrep. Several confounding time-series were calculated based on the preprocessed BOLD. The six head-motion estimates calculated in the correction step were included as confounding time-series, along with the temporal derivatives and quadratic terms. A set of physiological regressors were extracted to allow for component-based noise correction (Behzadi, Restom, Liau, & Liu, 2007). Principal components were estimated after high-pass filtering the preprocessed BOLD time-series (using a discrete cosine filter with a 128-s cutoff) based on anatomical masks (aCompCor). We used aCOMPCOR because it has proven to be one of the most consistent pipelines for mitigating the problem of motion across performance indices with dynamic functional connectivity analyses (Lydon-Staley, Ciric, Satterthwaite, & Bassett, 2019). Components were calculated within the intersection of the aforementioned mask and the union of CSF and white matter masks calculated in T1w space, after their projection to the native space of each functional run (using the inverse BOLD-to-T1w transformation). Components were also calculated separately within the white matter and CSF masks. *k* components with the largest singular values were retained, and the remaining components were dropped from consideration. All resamplings were performed with a single interpolation step by composing all the relevant transformations (i.e., head-motion transform matrices, susceptibility distortion correction when available, and co-registrations to anatomical and output spaces). Gridded (volumetric) resamplings were performed using antsApplyTransforms (ANTs), configured with Lanczos interpolation to minimize the smoothing effects of other kernels (Lanczos, 1964). Nongridded (surface) resamplings were performed using mri_vol2surf (FreeSurfer).

Many internal operations of fMRIPrep use Nilearn 0.5.2 (Abraham et al., 2014; RRID:SCR_001362), mostly within the functional processing workflow. For more details of the pipeline, see the section corresponding to workflows in fMRIPrep’s documentation.

#### Region of interest (ROI) signal extraction.

ROI extraction and nuisance regression were performed using Nilearn 0.5.2 (Abraham et al., 2014). For each participant, the raw BOLD time-series for each voxel inside a given ROI was isolated using the Schaeffer-100 atlas for cortical areas (Schaefer et al., 2018), the Harvard-Oxford atlas for subcortical areas (Desikan et al., 2006; Frazier et al., 2005; Goldstein et al., 2007; Makris et al., 2006), and the SUIT cerebellar atlas for the cerebellum (Jorn Diedrichsen, Balsters, Flavell, Cussans, & Ramnani, 2009). The mean time course of each ROI was high-pass filtered (cutoff = 0.01 Hz), temporally detrended, and *z*-scored across each run, in order to ensure that all voxels had the same scale of activation after preprocessing. In order to account for motion confounds and physiological noise, the above-described nuisance regressors were included, that is, participants’ six motion parameters and their derivatives, squares, and the squares of the derivatives (24 total) and the top six aCompCor regressors. Note that nuisance regression was performed orthogonally to temporal filters, so as to avoid reintroducing high-frequency noise from the regressors (Lindquist, Geuter, Wager, & Caffo, 2019).

### Data Analysis

#### Behavioral analyses.

To assess performance on the task, we calculated the angle of the cursor relative to the target at the moment the cursor reached the target distance (or the nearest data point if this value could not be used). This calculation was performed by subtracting the target angle from an angle derived from the horizontal and vertical position at a 300-pixel distance from the starting point. The median endpoint cursor error for the bin of eight trials (one trial for each target) was extracted (binned median error), and the mean binned median error for early trials (bins 1–3; 24 trials total) and late trials (bins 38–40; 24 trials total) were calculated to yield “early” and “late” error measures for all participants on each day. Savings was measured by subtracting the early error on Day 2 from the early error on Day 1 (Morehead et al., 2015). To determine whether distinct subgroups of learners were present in our sample, *k*-means clustering (Lloyd, 1982) was performed across participants using these five learning measures (early error on Day 1, early error on Day 2, late error on Day 1, late error on Day 2, and savings). We ran *k*-means until convergence 1,000 times with random initialization for all integers in 2 ≤ k ≤ 9 (maximum of 10,000 iterations of the algorithm for each initialization). The solution with the lowest sum of Euclidean distances between the data points and their assigned centroids was chosen for each value of k, and each of these solutions was evaluated by the Silhouette Rousseeuw (1987) and Caliński-Harabasz Caliński and Harabasz (1974) cluster evaluation indices. The solution corresponding to *k* = 3 had the highest score according to both indices (see Standage et al., 2023). Results were identical under Euclidean and Manhattan distances measures. We tested this clustering solution for statistical significance by randomly drawing from a multivariate normal distribution with the same means and covariance as the data (1,000 iterations, one draw for each participant), running *k*-means for each iteration in the same way as above. We recorded the highest value of the Silhouette and Calinski-Harabasz indices (over all k) for each iteration, where the probability of falsely rejecting the null hypothesis (for each evaluation index) was equated with the proportion of iterations with a higher score than our clustering solution.

#### Temporal modularity of functional networks.

Following earlier work (Bassett et al., 2011), the maximum overlap discrete wavelet transform was used to decompose the time-series for each ROI into wavelet coefficients in the range of 0.12–0.25 Hz (Haar wavelet family, scale 1). The decomposed time-series were divided into windows of w = 64 s (32 imaging volumes; corresponding to two bins of eight trials; see above) where contiguous windows overlapped by one bin, that is, by 50% (Chai, Mattar, Blank, Fedorenko, & Bassett, 2016; Standage et al., 2020). We constructed functional networks in each time window by calculating the magnitude squared spectral coherence between each pair of brain regions and taking the mean coherence over the specified range of frequencies (Bassett et al., 2011). All coherence values less than the 95th percentile of a null model were set to zero. For each of baseline, learning, and unlearning, the null model was constructed over 10,000 iterations by selecting two regions and start times at random, shuffling their contents over window w and calculating their coherence in the same way as for the original time-series. This approach produced multislice networks with a mean sparsity of ∼85% (i.e., 85% of connections were zero). We determined the modular composition of the resulting multislice networks with a generalized Louvain method for time-resolved clustering (Jeub, Bazzi, Jutla, & Mucha, n.d.). This algorithm was repeated 100 times, resulting in 100 clustering solutions (a.k.a. partitions), each of which maximized a modularity “quality” function (Mucha et al., 2010). On each repetition, we initialized the algorithm at random (uniform distribution) and iterated until a stable partition was found; that is, the output on iteration *n* served as the input on iteration *n* + 1 until the output matched the input (Bassett et al., 2011; Mucha et al., 2010) where nodes were assigned to modules at random from all assignments that increased the quality function, and the probability of assignment was proportional to this increase. We used the standard spatial and temporal resolution parameters *γ* = 1 and *ω* = 1, respectively, and the standard null model by Newman and Girvan (2004) for the quality function (Mucha et al., 2010; Sporns & Betzel, 2016).

#### Linear regression modeling.

Linear regression models were of the form *y* = *β*_0_ + Σ*_i_ β_i_x_i_* where dependent variable *y* = PC1 in all cases. In simple linear regression models (*i* = 1), predictor variables *x* refer to *Q*, cohesion strength, disjointedness, recruitment, integration and node strength. In the multiple regression model in the Results section “The Strength of Resting-State Functional Connectivity Predicted Learning Performance,” *i* = 5 and *x* refers to principle component scores derived from these network-based measures.

For simple linear regression, we determined the model’s internal validity with LOOCV, calculating the squared difference between the real and predicted values of the independent (predictor) variable for each participant when the model was trained on the other 31 participants’ predictor and dependent variables. We then determined the statistical significance of this performance metric by permuting the mapping between the predictor and dependent variables 10,000 times, producing a null distribution of (across-participant) mean squared errors. The model was considered significant if the (real) mean squared error was in the 95th percentile of the null distribution. All regression modeling was done with the statsmodels package (0.13.5; Seabold & Perktold, 2010) for Python (3.11.0).

#### Module allegiance matrix.

We constructed a matrix *T*, where the elements *T*_*i, j*_ refer to the number of times regions *i* and *j* were assigned to the same module over all time slices, partitions, and participants during early learning (wavelet scale 2; Standage et al., 2023) and (separately) the first resting scan. We then constructed the module allegiance matrix *P* = (1/*C*)*T*, where *C* is the total number of time slices in all of these partitions. Values that did not exceed those of a null model were set to 0 (Braun et al., 2015).

#### Clustering of the module allegiance matrix with nonnegative matrix factorization.

To derive summary networks from the module allegiance matrix, we used symmetric nonnegative matrix factorization (SymNMF; Kuang, Ding, & Park, 2012; Lee & Seung, 1999), which decomposes a symmetric matrix *Y* as *Y* = *AAT*, where *A* is a rectangular matrix containing a set of nonnegative factors that minimize a sum-of-squares error criterion. SymNMF has previously shown good performance in community detection problems (Kuang et al., 2012; Kuang, Yun, & Park, 2015) and has the benefit of producing a natural clustering solution by assigning each observation to the factor on which it loads most strongly. We used the Newton-like SymNMF algorithm described by Kuang et al. (2012), where for each number of factors between 2 and 15, we fit the model using 250 random initializations of the factor matrix H, with entries sampled from a uniform distribution on the interval [0, 1]. We then computed, for each rank, the average root mean squared reconstruction error, the dispersion coefficient (Kim & Park, 2007), and the cophenetic correlation (Brunet, Tamayo, Golub, & Mesirov, 2004). The latter two measures quantify the consistency of the clustering solutions returned by different initializations. We judged that a rank 5 solution was acceptable, as it explained over 80% of the variance in the observed data, it was acceptable by both the dispersion and cophenetic correlation criteria, and the generation of additional clusters provided little improvement in explained variance. We then selected the rank 5 solution with the lowest reconstruction error and generated a clustering solution by assigning each brain region to the factor with the highest loading.

## ACKNOWLEDGMENTS

This work was supported by the Canadian Institutes of Health Research and the Natural Sciences and Engineering Research Council of Canada.

## SUPPORTING INFORMATION

Supporting information for this article is available at https://doi.org/10.1162/netn_a_00420.

## AUTHOR CONTRIBUTIONS

Dominic I. Standage: Conceptualization; Data curation; Formal analysis; Funding acquisition; Investigation; Methodology; Project administration; Resources; Software; Validation; Visualization; Writing – original draft; Writing – review & editing. Daniel J. Gale: Data curation; Resources; Software; Validation. Joseph Y. Nashed: Data curation; Resources; Software; Validation. J. Randall Flanagan: Conceptualization; Funding acquisition; Methodology; Project administration; Resources. Jason P. Gallivan: Conceptualization; Data curation; Funding acquisition; Investigation; Methodology; Project administration; Resources; Supervision; Validation; Writing – review & editing.

## FUNDING INFORMATION

Jason P. Gallivan, Canadian institutes for health research, Award ID: PJT175012. Jason P. Gallivan, Natural Sciences and Engineering Research Council of Canada (https://dx.doi.org/10.13039/501100000038), Award ID: RGPIN-2017-04684.

## Supplementary Material



## TECHNICAL TERMS

fMRI: A slow, indirect measure of neural activity, with high spatial resolution.
Functional connectivity: The covariation of all pairs of brain signals over time, expressed as a network.
Modularity: Division of a network into subnetworks with strong within-subnetwork connectivity and weak between-subnetwork connectivity.
Integration: A way to quantify the degree of overlap between modules over time.
Modular reconfiguration: Changes in modular membership over time.
Dynamic modularity: When the identities of modules are preserved as their membership changes over time.
Recruitment: A way to quantify the stability of a module over time.
Quality function: The objective function assigning a score to a modular configuration.
Cohesion strength: The degree to which network nodes move between modules in groups, that is, coordinated reconfiguration.
Disjointedness: The degree to which network nodes move between modules by themselves, that is, uncoordinated reconfiguration.
