# Supplementary material for: Whole-brain modular dynamics at rest predict sensorimotor learning performance
Source: Netw Neurosci. 2025 May 8;9(2):631–60. doi: 10.1162/netn_a_00420 (PMC12140580; doi:10.1162/netn_a_00420)
Supplement: Supplementary file 1 [file netn-9-2-631-s001.pdf]

Whole-brain modular dynamics at rest predict sensorimotor learning performance SUPPLEMENTARY MATERIALS

Dominic I. Standage[1,2,✉], **Daniel J. Gale**[2], **Joseph Y. Nashed**[2], **J. Randall Flanagan**[2,3], **and Jason P. Gallivan**[1,2,3]

[1]Department of Biomedical and Molecular Sciences, Queen's University, Kingston Canada
[2]Centre for Neuroscience Studies, Queen's University, Kingston Canada
[3]Department of Psychology, Queen's University, Kingston Canada

Correspondence: ***standage@queensu.ca***

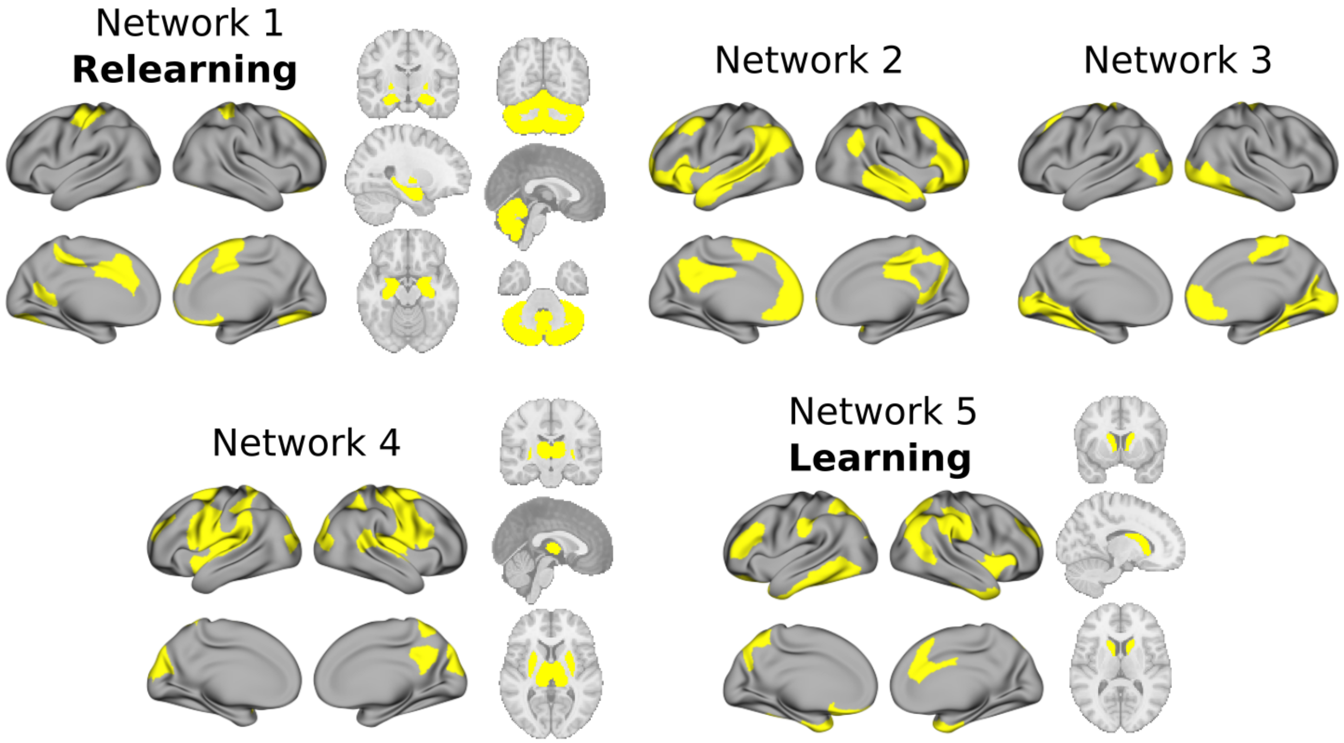

Fig. 1. Static networks summarising modular dynamics, derived during early learning by Standage et al. (1), rendered onto cortical and subcortical surfaces (areas in yellow denote the derived networks). Network 1 consisted of regions spanning contralateral motor cortex, bilateral cerebellum, medial prefrontal cortex and several subcortical structures (bilateral hippocampus, pallidum, amygdala and accumbens). This network is referred to as the relearning network (bold text) in the present work, due to its association with learning on the second day of testing (*i.e.* relearning, see main text). Network 2 was a purely cortical network, consisting of regions spanning angular gyrus, superior temporal gyrus, cingulate cortex and medial prefrontal cortex. Network 3 was also a purely cortical network, consisting mainly of regions in visual and fusiform cortex, and medial somatomotor cortex. Network 4 consisted of regions in visual cortex, medial and lateral parietal cortex, lateral somatomotor and premotor cortex, along with bilateral thalamus and putamen. Lastly, Network 5 consisted of regions spanning the anterior temporal pole, inferior and superior parietal, dorsolateral prefrontal cortex and the bilateral caudate. We refer to this network as the learning network (bold text) in the present work, due to its association with learning on the first day of testing.

Supplementary Note 1: Static networks summarising modular dynamics

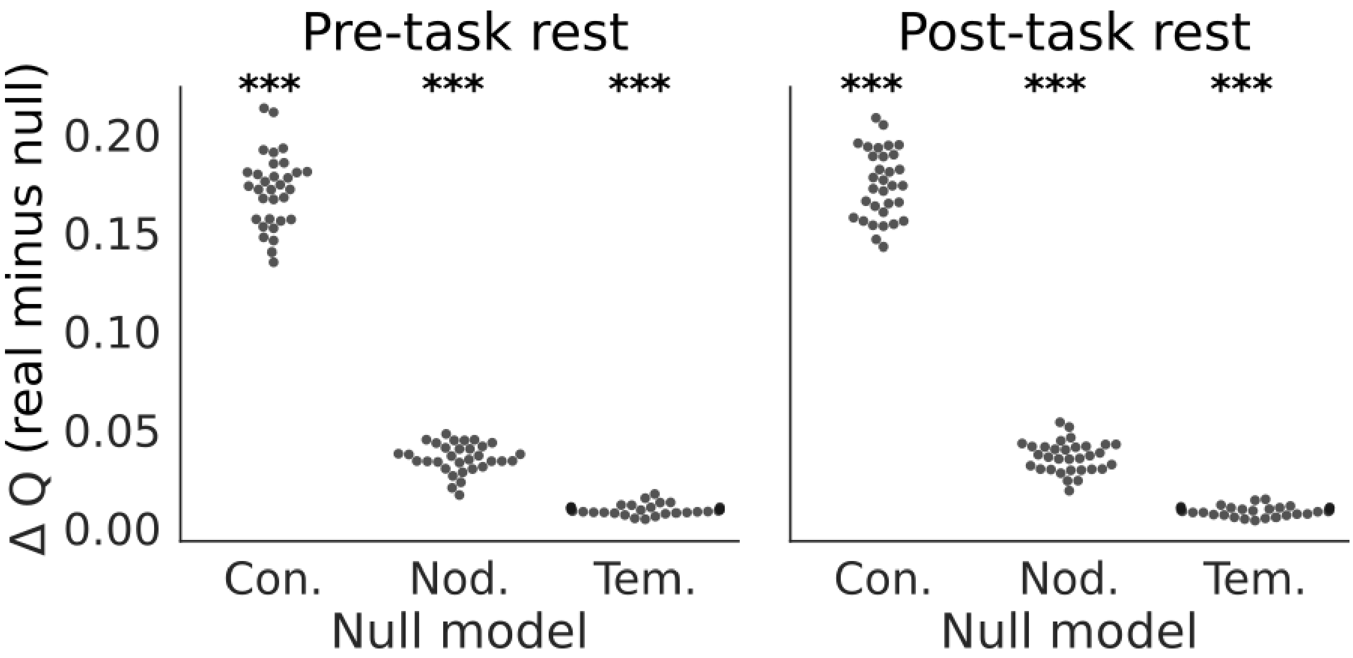

Fig. 2. Temporal networks were significantly modular at rest, before and after learning. Scatter plots show the difference (ΔQ) in the modular quality function Q between real and permuted networks (real minus permuted) for connectional (Con.), nodal (Nod.) and temporal (Tem.) null models (2) during pre-task (left) [connectional: t(31) = 52.493, p = 7.453e-31; nodal: t(31) = 27.271, p = 3.128e-23; temporal: t(31) = 19.807, p = 3.717e-19] and post-task (right) [connectional: t(31) = 56.447, p = 8.031e-33; nodal: t(31) = 26.082, p = 1.179e-22; temporal t(31) = 19.564, p = 5.315e-19] rest. Each dot corresponds to a participant. Jitter is for visual clarity. As in the main text, three stars indicate p < 1e-3.

Supplementary Note 2: Resting-state functional networks were significantly modular

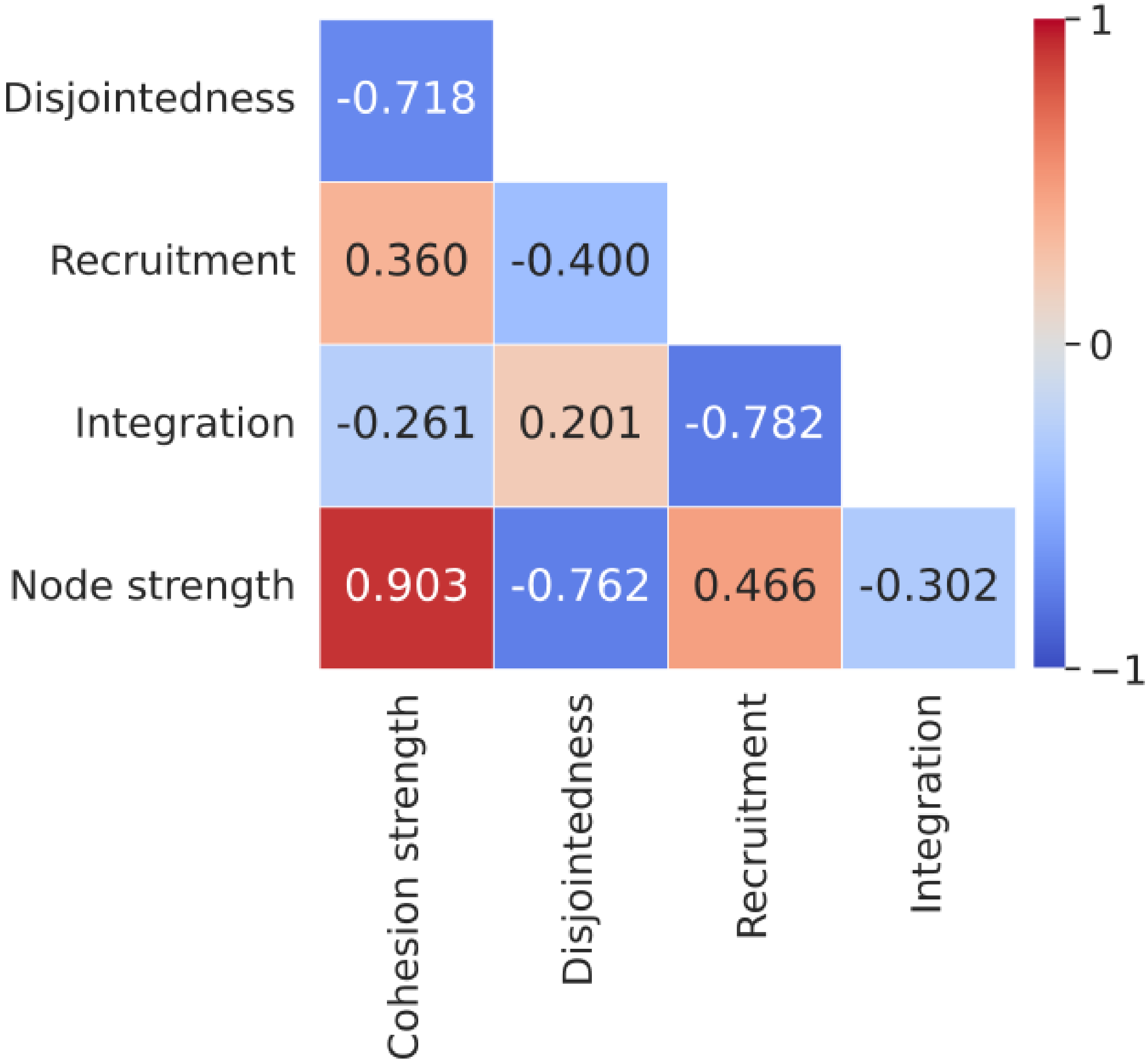

Fig. 3. Network-based variables that predicted participants' learning behaviour showed varying strengths of correlation. The strength of the Pearson correlation coefficient is colour coded from strongly negative (darkest blue) to strongly positive (darkest red) for all pairs of neural variables that predicted PC1, our proxy for group membership (see main text). The value of each correlation coefficient is also printed for each pair of variables.

Supplementary Note 3: Correlations between neural predictor variables

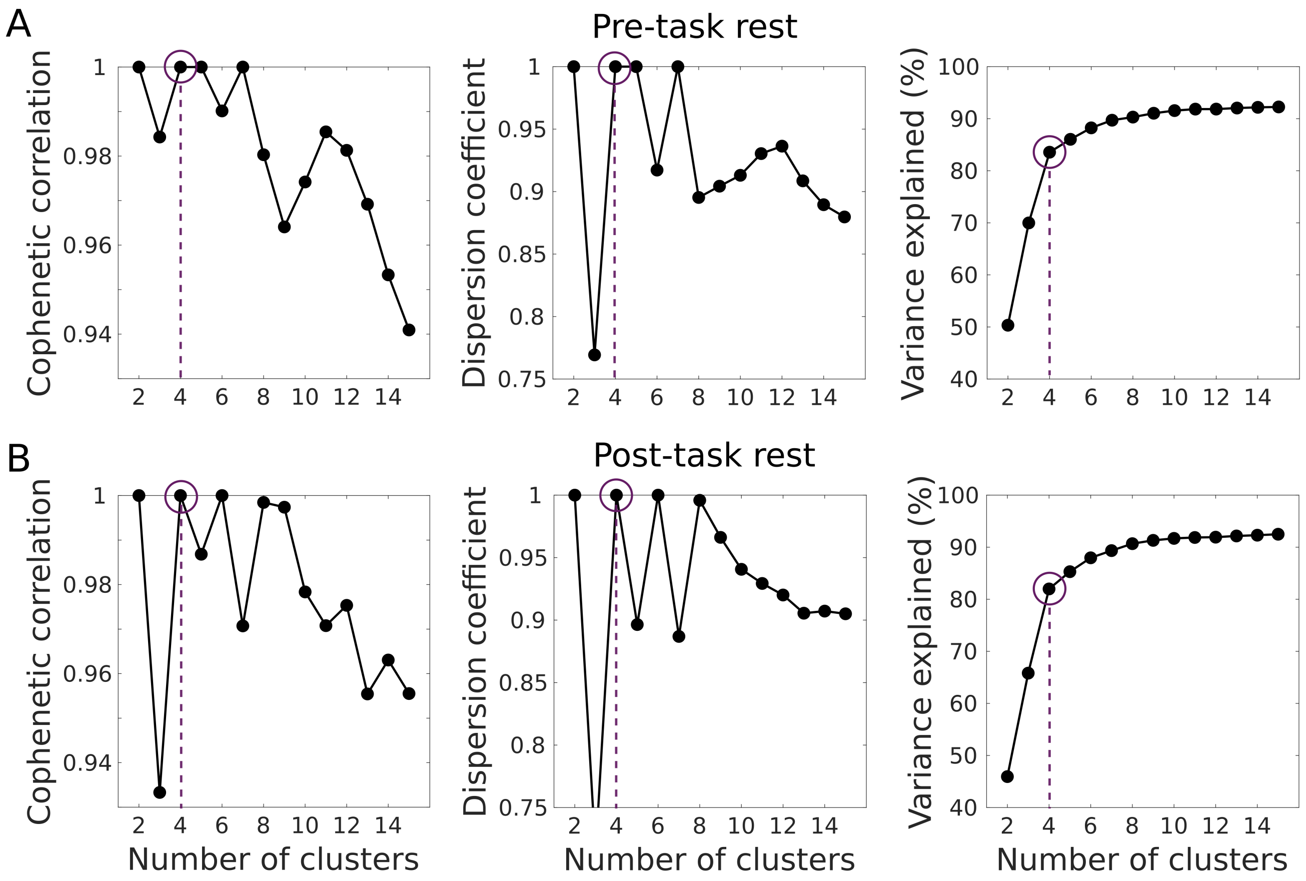

Fig. 4. Clustering diagnostics for symmetric non-negative matrix factorization of pre- and post-task module allegiance matrices. Symmetric NMF was performed using 250 random initializations for factors 2 to 15. Regions were then assigned to a cluster corresponding to the factor with the greatest loading. For each number of factors, we computed the cophenetic correlation, dispersion, and explained variance. Dashed vertical line in each panel indicates the chosen solution of 4 clusters for pre-task (A) and post-task (B) rest.

Supplementary Note 4: Clustering diagnostics identified four resting-state networks during pre- and post-task rest

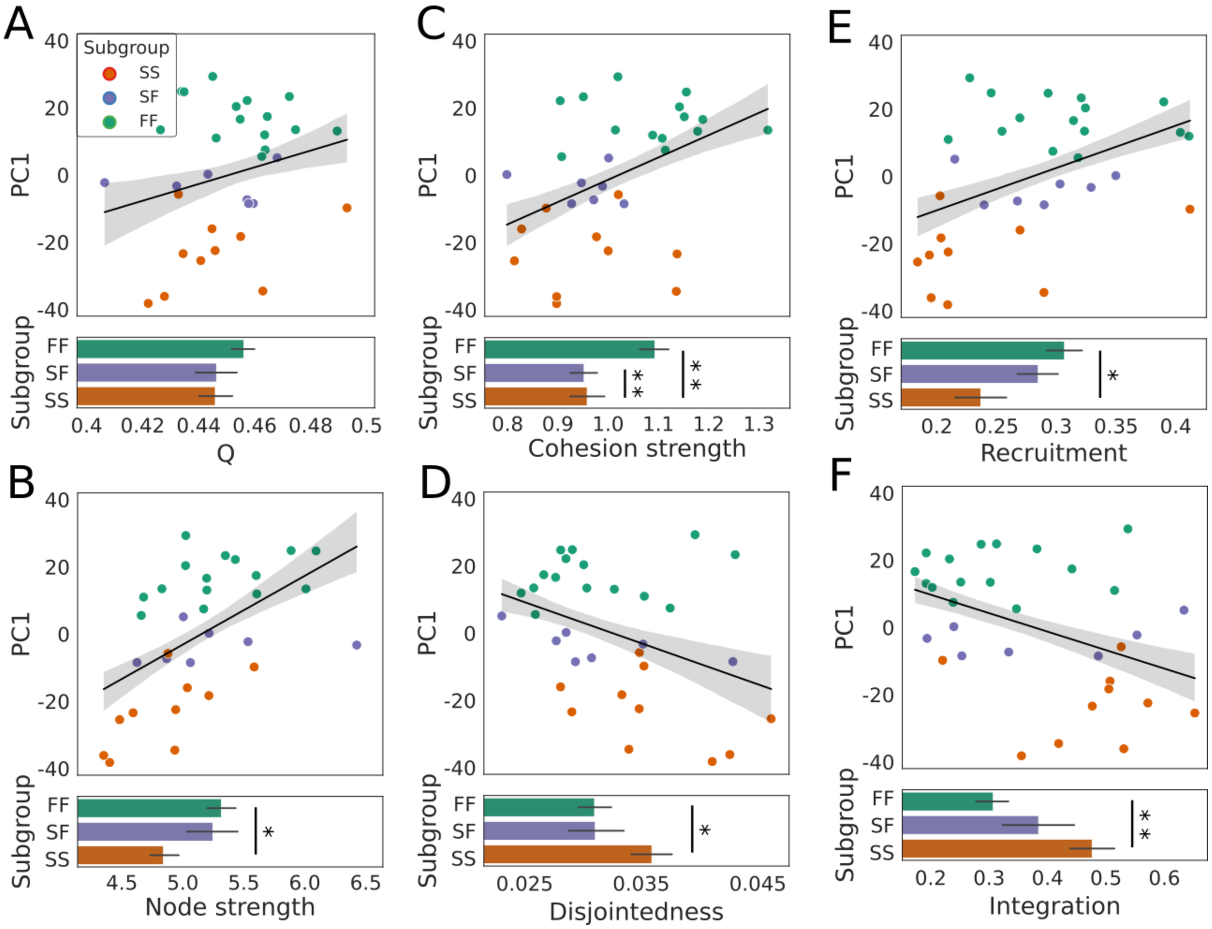

Fig. 5. Neural predictions of behaviour were qualitatively unchanged with uniformly sparse networks (90% sparse). (A) As in the main text, pre-task dynamic modularity scores did not predict participants' learning profiles. The scatter plot shows PC1 for each subject (our proxy for behavioural subgroup membership) as a function of the mean quality function score Q. Filled circles correspond to the FF, SS, and SF behavioural subgroups (see legend). The fitted line (black) shows a linear regression model (least squares fit) where the shaded area corresponds to ± 1 standard error (SE). Subgroup means ± 1 SE are shown as bar graphs below the scatter plots. The fit was non-significant [R^2 = 0.060, F(1,30) = 1.931, p = 0.175]. **(B)** As determined by a linear regression model, pre-task node strength at rest predicted PC1 [R^2 = 0.281, F(1,30) = 11.720, p = 0.002], where FF was significantly stronger than SS [two-sample t-test: t(23) = 2.704, p = 0.013], but SF did not differ statistically from FF [t(20) = -0.303, p = 0.765] or SS [t(15) = 1.712, p = 0.107]. The model was internally validated by permutation testing with leave-one-out cross validation (LOOCV, see main text) [mean squared error = 307.228, standard deviation = 342.155, p = 0.005]. **(C)** Pre-task cohesion strength predicted PC1 [R^2 = 0.185, F(1,30) = 6.828, p = 0.0139], where FF was more cohesive than SS [t(23) = 2.870, p = 0.009] and SF [t(20) = 2.951, p = 0.008] but SS and SF did not differ statistically [t(15) = -0.122, p = 0.905]. The linear regression model was validated by permutation testing with LOOCV [mean squared error = 332.734, standard deviation = 462.226, p = 0.012]. **(D)** Pre-task disjointedness was negatively predictive of PC1 [R^2 = 0.145, F(1,30) = 5.088, p = 0.032], where FF was significantly more disjointed than SS [t(23) = -2.149, p = 0.042], but SF did not differ statistically from FF [t(20) = -0.025, p = 0.980] or SS [t(15) = -1.643, p = 0.121]. The model was validated by permutation testing with LOOCV [mean squared error = 359.131, standard deviation = 451.440, p = 0.044].**(E)** Pre-task recruitment of the relearning network at rest predicted PC1 [R^2 = 0.191, F(1,30) = 7.089, p = 0.012, FDR-adjusted p: ???], where recruitment by FF was stronger than by SS [t(23) = 2.643, p = 0.015], but recruitment by SF did not differ statistically from that by FF [t(20) = 0.839, p = 0.412] or SS [t(15) = 1.559, p = 0.140]. The model was validated by permutation testing with LOOCV [mean squared error = 359.131, standard deviation = 394.485, p = 0.012]. **(F)** Pre-task integration of the learning and relearning networks was (negatively) predictive of PC1 [R^2 = 0.175, F(1,30) = 6.350, p = 0.017], where integration was stronger by the SS subgroup than FF [t(23) = 3.509 , p = 0.002] but did not differ statistically between SF and FF [t(20) = 1.2505, p = 0.226] or SS [t(15) = 1.295, p = 0.215]. The model was validated by permutation testing with LOOCV [mean squared error = 335.775, standard deviation = 429.836, p = 0.015]. In bar plots in all panels, error bars show ± 1 SE, where one ($p < 0.05$) and two ($p < 0.01$) stars indicate significant differences.

Supplementary Note 5: Predictions were replicated with uniformly sparse networks

Supplementary bibliography

1. Dominic I. Standage, Corson N. Arshenkoff, Daniel J. Gale, Joseph Y. Nashed, J. Randall Flanagan, and Jason P. Gallivan. Whole-brain dynamics of human sensorimotor adaptation. *Cerebral cortex*, bhac378:1–18, 2022.
2. Peter J. Mucha, Thomas Richardson, Kevin Macon, Mason A. Porter, and Jukka-Pekka Onnela. Community structure in time-dependent, multiscale, and multiplex networks. *Science*, 328:876–878, 2010.
